# Gene regulatory network inference based on causal discovery integrating with graph neural network

**DOI:** 10.1002/qub2.26

**Published:** 2023-12-22

**Authors:** Ke Feng, Hongyang Jiang, Chaoyi Yin, Huiyan Sun

**Affiliations:** ^1^ School of Artificial Intelligence Jilin University Changchun China; ^2^ International Center of Future Science Jilin University Changchun China; ^3^ Engineering Research Center of Knowledge‐Driven Human‐Machine Intelligence Ministry of Education Changchun China

**Keywords:** causal discovery, ensemble learning, gene regulatory network inference, gene regulatory networks, graph neural network, key regulators of disease development

## Abstract

Gene regulatory network (GRN) inference from gene expression data is a significant approach to understanding aspects of the biological system. Compared with generalized correlation‐based methods, causality‐inspired ones seem more rational to infer regulatory relationships. We propose GRINCD, a novel GRN inference framework empowered by graph representation learning and causal asymmetric learning, considering both linear and non‐linear regulatory relationships. First, high‐quality representation of each gene is generated using graph neural network. Then, we apply the additive noise model to predict the causal regulation of each regulator‐target pair. Additionally, we design two channels and finally assemble them for robust prediction. Through comprehensive comparisons of our framework with state‐of‐the‐art methods based on different principles on numerous datasets of diverse types and scales, the experimental results show that our framework achieves superior or comparable performance under various evaluation metrics. Our work provides a new clue for constructing GRNs, and our proposed framework GRINCD also shows potential in identifying key factors affecting cancer development.

## 1 INTRODUCTION

Gene regulatory networks (GRNs) characterize the regulatory mechanism of genes in a form of a network in the cell system [1]. This observation provides an essential clue for us to comprehend cell procedures and molecular interplay that govern cell phenotypes. Transcriptional regulation is a common means of regulation of gene expression, in which the target genes (TGs) are regulated by transcription factors (TFs). The development of high‐throughput technologies has produced tremendous amounts of gene expression data, which provide an important basis for underlying regulatory mechanisms. After decades of effort, many computational methods have been developed to infer GRN from observational gene expression data [2, 3]. However, although the regulatory mechanism is intrinsically causal, most methods are limited to correlation analysis.

Generalized correlation‐based methods are mainly divided into the following categories: regression‐based, information theory‐based, Bayesian network‐based, ordinary differential equation (ODE)‐based, etc. Regression‐based methods usually regress the gene expression of a TG on a set of TFs and take the regression coefficients as the regulatory strength between TFs and TGs [4, 5]. These methods mainly take improving regression accuracy of fitness as the object and are largely influenced by the number of samples and dependent variables. Compared to regression, correlation‐based methods, such as Pearson correlation, mutual information (MI)‐based CLR [6], and ARACNE [7] method, a Bayesian network [8] method, such as catnet [9] and max‐min parent and children algorithm [10], infers dependence among TFs and TGs by computing conditional independence among genes. Although Bayesian networks can reveal the interrelations within a complex multivariable system, the increase in time cost with the growing number of genes and strong constraints make these methods unsuitable for large‐scale gene networks. Also, Bayesian networks describe distribution over observed variables but don’t reveal what will happen if a certain intervention occurs, which causal networks do [11].

Although time labels/series data are admittedly important information for regulatory relationship determination, and are especially necessary for ODE‐based methods, there are few available biometric data with real‐time tags and appropriate time intervals. Although single cells can be labeled with pseudo time and RNA velocity information from single‐cell RNA‐seq (scRNA‐seq) data, recent studies have shown that neither of them performs as well as true time‐series data, so single‐cell pseudo time can hardly be used for ODE models [12]. Moreover, there are other classic machine learning‐based GRN inference methods, such as GENIE3 [13], which builds a random tree for each TG and ensembles the trees as a random forest to infer GRN by solving several independent regression problems, and TIGRESS [14] which incorporates Lasso and bootstrap to select 5 TFs for each TG. Considering prior network knowledge for the construction of a new GRN, some advanced methods based on deep learning models, such as 3D convolutional neural network (CNN) [15] and GRGNN [16], have been applied in previous studies.

From another perspective, as mentioned above, there is essentially a causal regulatory pathway from TFs to TG; causality‐based GRN inference methods seem more rational than generalized correlation‐based ones. Existing computational causality discovery methods mainly include time tag‐based ones and time tag‐free ones. The former rely on the notion that changes of outcome precede the intervention in time, such as Granger causality (GC) [17] and convergent cross mapping (CCM) [18] methods. Also due to the limitation of lack of time series data, CCM, which learns causal structure from observation data without time tags, is more applicable to the real scenario. Current causal discovery methods are usually classified into constraint‐based methods and functional causal models. The PC algorithm (named after its authors, Peter and Clark) [19] is a classic causal discovery method that first constructs a causal skeleton and then orients causal direction via conditional independence and constraints. On the basis of PC, many improved causal structure learning methods have been developed, such as DCI [20], IDA [21], and fast causal inference algorithm [19]. However, constraint‐based causality methods are also time‐consuming when it comes to large‐scale variables. Functional causal models have been proposed from the perspective of the data distribution characteristics generated by the causal mechanism and are usually joined with structural equation models. On this basis, learning a faithful directed acyclic graph (DAG) from observation data is an important part of causal discovery. NOTEARS [22] is such a functional causal model, with directed acyclic structure as the constraint, and is able to model the whole graph. However, it can’t be directly transferred to the GRN task as the DAG assumption is too strong for GRN inference [23].

In comparison, pairwise causal direction identification is another type of functional‐based causal discovery method. Such methods include the additive noise model (ANM) [24] and the post non‐linear model (PNL) [25]. These methods relax the assumption of DAG and seem more straightforward and reasonable for constructing GRN, by calculating causal directions for all potential TF‐TG and TF‐TF pairs. Moreover, in biologically complex systems where each molecule does not work independently, identifying causal direction between two genes only based on their isolated gene expression is unreliable. Hence, it is necessary to aggregate the information associated with the two genes in the system to represent them. Graph neural network (GNN) is a powerful deep learning model for graph structure data, and has good representation learning ability. Inspired by the convolution operation of the CNN [26] for grid‐like data, graph convolutional network (GCN) [27], one of the most popular GNN algorithms, uses the convolution operation to aggregate neighbors’ information and then to update representation of nodes. GraphSAGE [28] improves conventional GCN by sampling and aggregating information of neighbors to learn node embedding with node features, making it applicable for representation of nodes in large graphs to solve the problems of memory explosion and inflexibility of GCN together.

In this paper, we propose GRINCD, a GRN inference framework based on pairwise causal discovery, which uses both gene expression and gene representation derived from the GNN model. Concretely, we first construct a potential gene co‐expression network and then generate each gene’s new representation composing both gene expression and gene representation. This representation is derived from information aggregation of connected nodes in the system through training a GraphSAGE model. Finally, we develop an ANM‐based method to calculate pairwise causal regulatory relationships for all the TF‐TG and TF‐TF pairs. It is worth noting that considering both the linear and non‐linear relationships, we take different adaptive approaches to constructing a linear pipeline and a non‐linear pipeline, covering the above steps, and eventually integrate them together. We conduct a series of experiments on transcriptomic data of model organisms and human single‐cells to evaluate the performance of GRINCD. Compared with various types of methods under different metrics, GRINCD achieves superior or comparable performance in predicting the regulatory relationship of not only TF‐TG but also TF‐TF for which generalized correlation‐based methods are unattainable. In addition, when applying the GRINCD model to infer various and crucial regulatory relationships that are important during the transition from inflammatory bowel disease to colorectal cancer (COAD), the model reveals that a certain bunch of TFs, but not all, provide the driving force of cancer development. GRINCD fills the gap of effective causal‐based GRN inference on large‐scale datasets and leads causal discovery to broader biological application.

## 2 RESULTS

### 2.1 Overview of GRINCD

**FIGURE 1:**
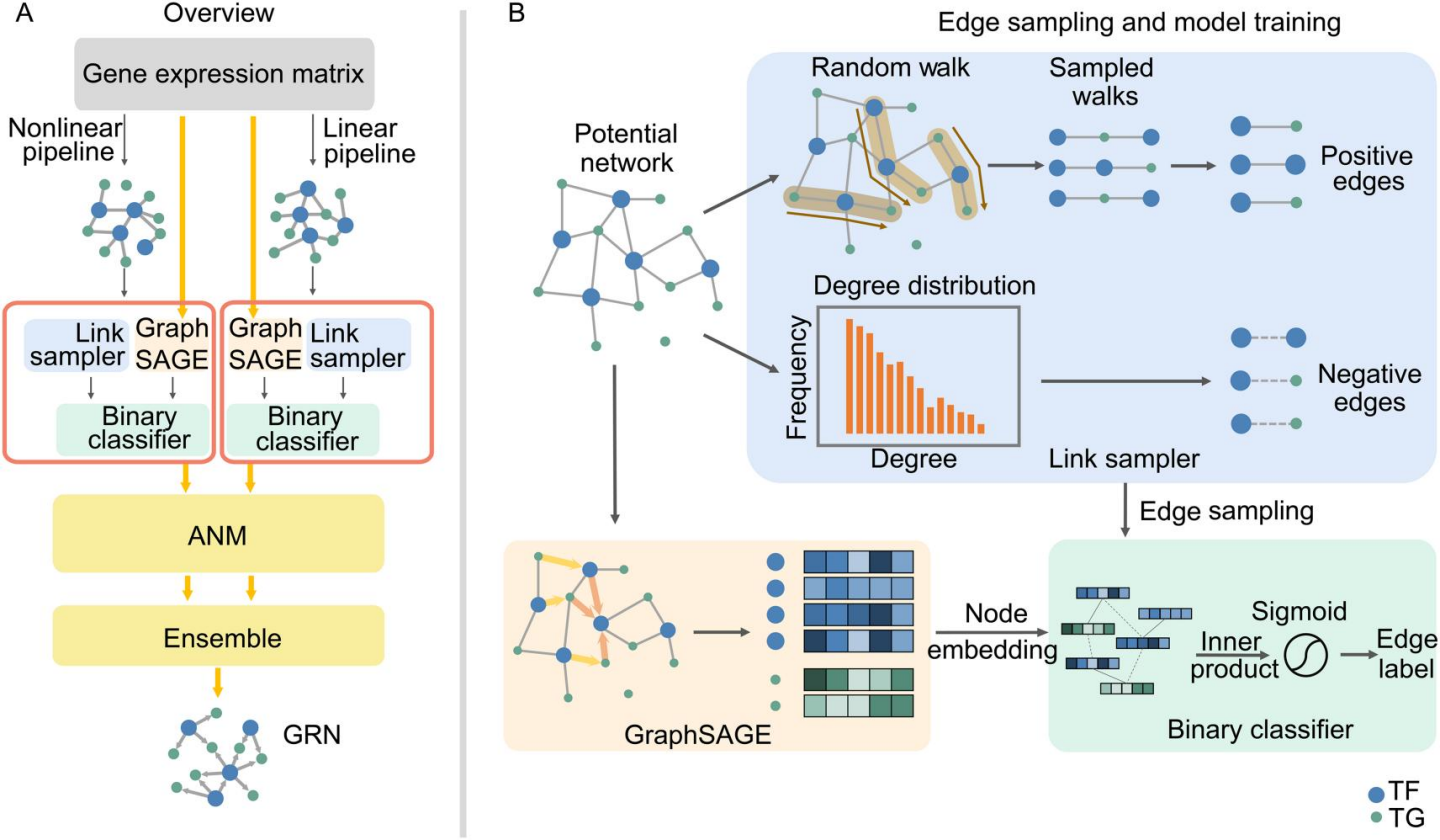
The GRINCD framework. (A) The GRINCD framework includes three steps: Step 1 is to construct potential networks from gene expression data, where edges represent strong correlations between genes. Step 2 is training GraphSAGE with potential network from step1 to obtain the new representation of each gene, a binary classifier is used as the downstream task to guide the model training, taking high‐quality representation for each gene. Step 3 infers causal regulatory relationships by applying the additive noise model (ANM) model. We separately employ a linear pipeline and a nonlinear pipeline, and then finally integrate them to infer Gene regulatory network (GRN). (B) Link Sampler utilizes random walk and degree distribution of nodes to generate positive edges and negative edges. We sample the same number of positive and negative edges for model training. After edge sampling, we feed these samples to a two‐layer GraphSAGE connected with a binary classifier to train the whole neural network (NN). GraphSAGE is well‐trained when the binary classifier gets high accuracy.

GRINCD is designed with a gene expression matrix as input, and a ranked gene pair list, sorted decreasingly by the corresponding possibility, as output. There are three main steps in this framework, as shown in Figure 1A: (i) a potential network is constructed from gene expression data, where an edge represents a strong correlation between genes, (ii) we sample positive and negative edges from the potential network and then train GraphSAGE to obtain the new representation of each gene by using binary classification as the downstream task, (iii) genes’ representation from the well‐trained GraphSAGE is fed into modified ANM to infer causal regulatory relationships. Since the regulatory relationship between genes may be linear or nonlinear, we employ two pipelines across these three steps to represent linear and nonlinear regulatory mechanisms. These two pipelines act independently and are then integrated together to infer GRN.

### 2.2 Comparison of methods performance

**FIGURE 2:**
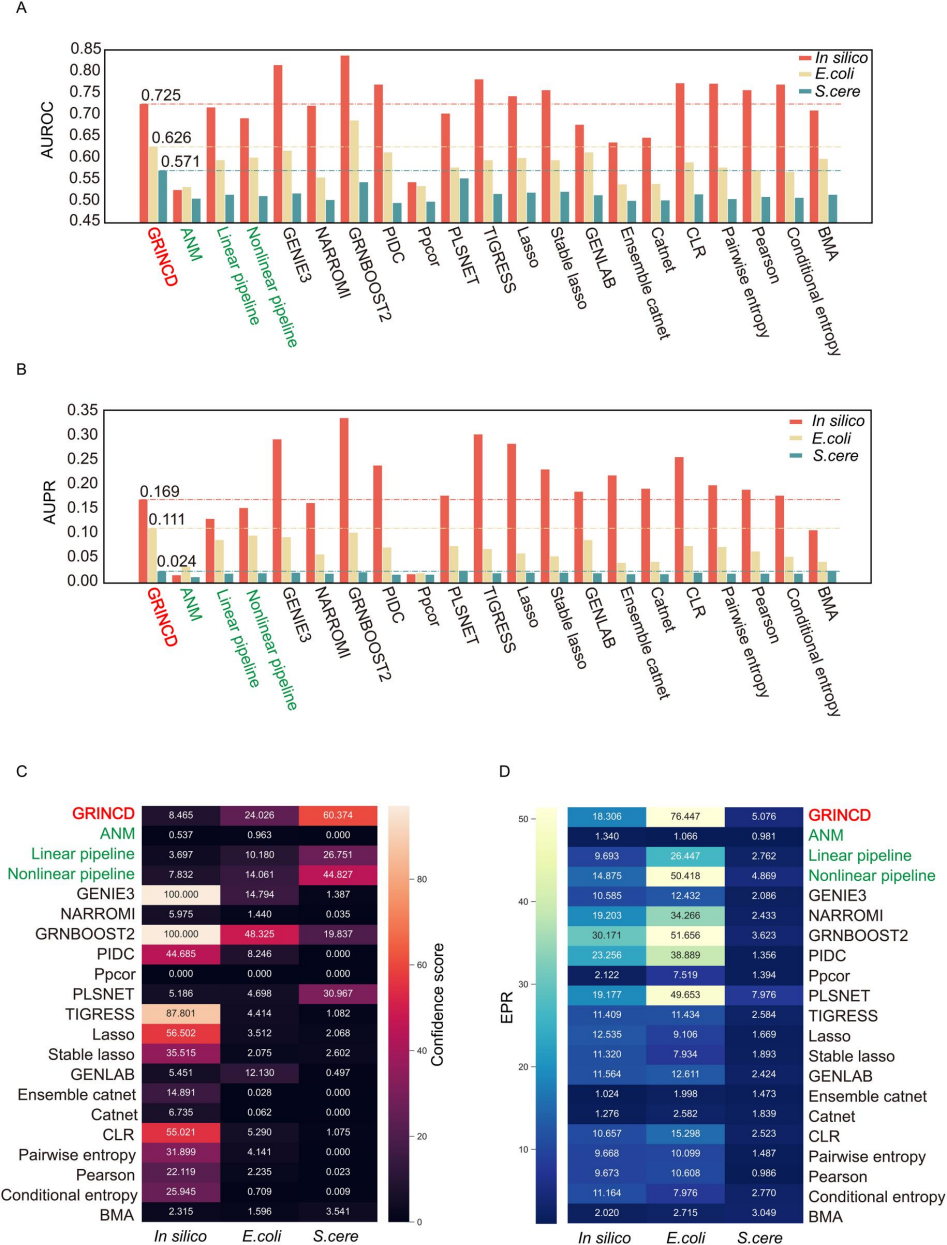
Summary of the gene regulatory network (GRN) prediction performance on DREAM5 datasets under different evaluation metrics. The dotted lines represent the performances of GRINCD, and the methods highlighted in green represent the results of ablation experiments. (A) Performance under AUROCs metric. (B) Performance under AUPRs metric. (C) Performance under confidence scores metric. (D) Performance under EPRs metric.

To evaluate the performance of various GRN inference methods, we collect three datasets from DREAM5 [1] and two scRNA‐seq datasets, one for mouse dendritic cells (mDC) and the other for human embryonic stem cells (hESC). In addition, for scRNA‐seq datasets, we take three different kinds of gold standards (STRING, non‐specific CHIP‐seq, and cell‐type‐specific CHIP‐seq) and use two different parameters to process raw data, as shown in Table 1 (see materials and methods). We make a comprehensive comparison of our framework with several methods including regression‐based methods, correlation‐based methods, and Bayesian network‐based methods. Overall, the performance of our framework is better than most of the baseline methods on various datasets in terms of the area under the receiver operating characteristic (AUROC), the area under the precision‐recall curve (AUPR), the confidence score, and the early precision ratio (EPR) metrics, as shown in Figure 2, Figures S1 and S2.

**TABLE 1:** Properties of datasets.

Dataset name	#Samples	#Genes	#TFs	#Edges in gold standard	Avg out‐degree	Avg in‐degree	#Isolated genes	#Isolated TFs
In silico	805	1643	195	4012	20.57	2.44	145	17
*Escherichia coli*	805	4511	334	2066	6.19	0.46	3512	193
*Saccharomyces cerevisiae*	536	5950	333	3940	11.83	0.66	4016	219
hESC_CTS_500	758	910	34	4545	133.68	4.99	95	0
hESC_CTS_1000		1410	34	7084	208.35	5.02	150	
hESC_NS_500		910	283	3441	12.16	3.78	157	
hESC_NS_1000		1410	292	4617	15.81	3.27	272	
hESC_STR_500		910	343	4257	12.41	4.68	399	
hESC_STR_1000		1410	351	5149	14.67	3.65	715	
mDC_CTS_500	383	821	20	756	37.80	0.92	378	0
mDC_CTS_1000		1321	21	1193	56.81	0.90	637	
mDC_NS_500		821	250	3067	12.27	3.74	187	
mDC_NS_1000		1321	254	3918	15.43	2.97	352	
mDC_STR_500		821	264	4815	18.24	5.86	342	
mDC_STR_1000		1321	273	5898	21.60	4.46	657	

From Figure 2A,B, we compare GRINCD with other 17 benchmark methods under AUROC and AUPR: (i) for in silico data, GRINCD outperforms 7 (41%) benchmarks on AUROC, 3 (18%) benchmarks on AUPR; (ii) for *Escherichia coli* data, GRINCD outperforms 16 (94%) benchmarks on AUROC and all (100%) benchmarks on AUPR; (iii) for *Saccharomyces cerevisiae* data, GRINCD outperforms 16 (94%) benchmarks on AUROC and all (100%) benchmarks on AUPR. From Figure 2C,D, when evaluated by confidence score and EPR: (i) for in silico data, GRINCD outperforms 6 (35%) benchmarks on confidence score, 13 (76%) benchmarks on EPR; (ii) for *E.coli* data, GRINCD outperforms 16 (94%) benchmarks on confidence score, all (100%) benchmarks on EPR; (iii) for *S.cere* data, GRINCD outperforms all (100%) benchmarks on confidence score and 16 (94%) benchmarks on EPR. Compared with AUROC and AUPR, the performances of various methods under confidence score and EPR show more significant variance. Overall, for the DREAM5 dataset, GRINCD performs better on *E.coli* and *S.cere* datasets than in silico dataset. A fundamental assumption of most benchmark methods is that mRNA levels of TFs and TGs tend to be correlated. However, this is correct for in silico, but maybe not for *E.coli* and *S.cere*. So, these methods will perform better on the in silico dataset. But GRINCD is calculated based on causality, and it can handle real‐world data more accurately, so GRINCD performs better on *E.coli* and *S.cere* than on in silico dataset. The accuracy of gold standards may also play a key role; the lower correlation of the mRNA level in real‐world datasets is mainly due to the complexity of increased regulatory relationships and the prevalence of post‐transcriptional regulation [1]. For simulated data, it is relatively simple to generate a comprehensive expression relationship between a small number of data, such as 10 or 100 genes, but when the number of genes is too large, such as thousands, this expression may be somewhat distorted.

In addition, the DREAM5 network inference challenge has another gene expression data *Staphylococcus aureus*. However, the dataset of *S.aureus* doesn’t contain sufficient validated interactions which makes it difficult and unsuitable to evaluate the performance of each method using this dataset. In order to ensure the integrity of the experiment, we still use the dataset of *S.aureus* provided by DREAM5 to evaluate various methods, but do not make a comparison (Table S1).

We also evaluate GRINCD on scRNA‐seq data of mDC and hESC. As shown in Figures S1 and S2, when compared with other 17 state‐of‐the‐art methods under AUROC and AUPR, with three different kinds of gold standards: (i) for mDC data, GRINCD achieves 2 (17%) best prediction results, 4 (34%) second‐best results, and 2 (17%) third‐best results; (ii) for hESC data, GRINCD achieves 7 (58%) best prediction performance, 2 (17%) second‐best results, and 3 (25%) third‐best results. Similarly, when evaluated under confidence score and EPR: (i) for mDC data, GRINCD achieves 3 (25%) second‐best results, and 3 (25%) third‐best results; (ii) for hESC data, GRINCD achieves 3 (25%) best prediction results, 5 (42%) second‐best results, and 4 (33%) third‐best results.

In our framework, we suggest that the ensemble of two parallel pipelines as well as gene representation are essential for GRN inference. To demonstrate this, we conduct ablation studies on the linear pipeline, the nonlinear pipeline, and node embedding to show their respective contributions. ANM alone performs like a random predictor, but ANM with node embedding improves the performance with averages of 0.119, 0.081, 30.455, and 32.147 compared, respectively, with ANM alone under AUROC, AUPR, confidence score, and EPR metrics, respectively in DREAM5 datasets. Similarly, ANM with node embedding improves the performance with averages of 0.042, 0.055, 17.453, and 1.456 under AUROC, AUPR, confidence score, and EPR metrics, respectively, in single‐cell datasets. We also assess the contribution of the ensemble strategy, and the average improvement of our framework is 0.035 for AUROC, 0.017 for AUPR, 13.064 for confidence score, and 15.099 for EPR toward the DREAM5 datasets, respectively. For single‐cell datasets, it improves the performance at the average of 0.032 for AUROC, 0.009 for AUPR, 6.671 for confidence score, and 0.705 for EPR.

### 2.3 Consistency in TF‐TF evaluation

As prior information on some TFs is well known, most correlation‐based methods work well for TF‐TG prediction without considering the true regulatory direction. However, regarding the regulation relationship from 1 TF to another TF, these methods are clearly powerless as they can’t ensure direction. In comparison, the proposed GRINCD method has obvious advantages.

When taking TFs information as input as prior knowledge, it undoubtedly improves GRN inference’s performance by excluding some nonexistent edges. The main aim of our framework is to predict causal directions for all possible gene pairs (TF‐TF and TF‐TG), assuming we don’t know which one is TF, and to verify whether the prediction of this causal direction is correct. Especially, for TF‐TF pairs whose causal directions are usually unknown in advance, our proposed method has the advantage to infer the regulatory direction of TF‐TF pairs in GRN. We use the same benchmark datasets and methods as above and only evaluate TF‐TF pairs this time. As shown in Figure S3, for DREAM5 datasets, when compared with other 17 benchmark methods under AUROC and AUPR: (i) for in silico data, GRINCD outperforms 7 (41%) benchmarks on AUROC, 3 (18%) benchmarks on AUPR; (ii) for *E.coli* data, GRINCD outperforms 14 (82%) benchmarks on AUROC, and 14 (82%) benchmarks on AUPR; (iii) for *S.cere* data, GRINCD outperforms 16 (94%) benchmarks on AUROC, and 16 (94%) benchmarks on AUPR. When evaluated by confidence score and EPR: (i) for in silico data, GRINCD outperforms 4 (24%) benchmarks on confidence score and 13 (76%) benchmarks on EPR; (ii) for *E.coli* data, GRINCD outperforms 15 (88%) benchmarks on confidence score, and 15 (88%) benchmarks on EPR; (iii) for *S.cere* data, GRINCD outperforms 16 (94%) benchmarks on confidence score and 16 (94%) benchmarks on EPR. As for scRNA‐seq data of mDC and hESC (Figures S4 and S5). When compared with other 17 state‐of‐the‐art methods under AUROC and AUPR with three different kinds of gold standards: (i) for mDC data, GRINCD achieves 1 (8%) best prediction results, 3 (25%) second‐best results, and 2 (17%) third‐best results; (ii) for hESC data, GRINCD achieves 5 (42%) best prediction performances, 2 (17%) second‐best results, and 1 (8%) third‐best results. Similarly, when evaluated under confidence score and EPR: (i) for mDC data, GRINCD achieves 1 (8%) second‐best result, and 2 (17%) third‐best results; (ii) for hESC data, GRINCD achieves 9 (75%) best prediction results and 1 (8%) second‐best result. Unlike in the case of the evaluation of all genes, the performances of all the methods (including GRINCD) show no significant differences.

### 2.4 Analysis of the top‐k regulatory relationships

In addition to using metrics such as AUROC for comparison among various methods, we also conduct two cross‐comparisons about the performance of different methods. One is the intersection of top‐k regulatory relationships between GRINCD and other methods, and the other is the accuracy of top‐k regulatory relationships for various methods including GRINCD.

**FIGURE 3:**
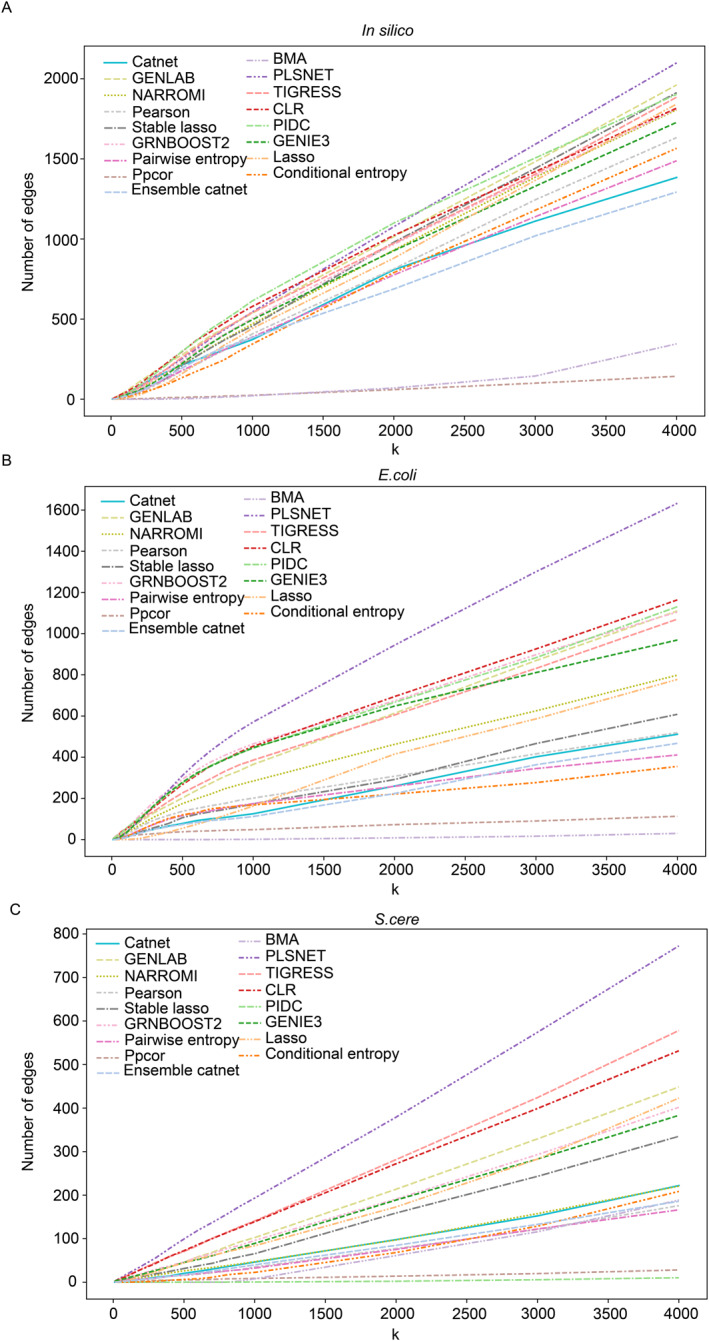
The intersections of top‐k regulatory relationships between GRINCD and other methods on DREAM5 datasets. (A) The intersection on the in silico dataset. (B) The intersection on the *E.coli* dataset. (C) The intersection on the *S.cere* dataset.

As shown in Figure 3, for DREAM5 datasets, the intersection comparison between GRINCD and various methods shows that GRINCD has more regulatory relationship intersections with methods of good performance. Some methods that have lower performance, such as ppcor and Bayesian model averaging algorithm (BMA), show significant differences from other methods. This experiment for single‐cell datasets is shown in Figures S6 and S7.

As for the accuracy rate of top‐k regulatory relations, we take a series of *k* values as shown in Tables S2–S4. For some methods, such as BMA, the result performs large differences on different datasets and different *k* values. BMA, for example, does not perform particularly well in terms of overall metrics, but on *S.cere*, it still gets the highest accuracy rate for some *k* values. The results of the comparison for single‐cell datasets are shown in Tables S4–S16.

For complex systems, using correlation to analyze the relationship between variables will increase a lot of false positives, but using causal‐based methods can solve this problem well. In the top‐k regulatory relationship accuracy experiment, we also compared the difference between GRINCD and correlation‐based methods. In the cross‐comparison of the performance experiment, we used two correlation‐based methods, ppcor, and Pearson. Compared with Pearson, GRINCD reduces the false‐positive rate (FPR) by 3.7%; on in silico data the improvement is 5.7%; for the *E.coli* dataset; and 0.2% for the *S.cere* dataset. For hESC datasets, GRINCD reduces the FPR by 2.7% on average, and by an average of 8.6% for mDC datasets. Compared with ppcor, GRINCD reduces the FPR by an average of 50.1% for the in silico dataset, 10.3% for the *E.coli* dataset, and 1.1% for the *S.cere* dataset. For hESC datasets, GRINCD reduced the FPR by an average of 4.8%, and for mDC datasets, GRINCD decreased the FPR by an average of 6.7%.

### 2.5 Performance from different sparsities of the potential network

As shown in Figure 2, Figures S1 and S2, ANM with gene representation shows much better performance than ANM alone, which means that high‐quality node embedding based on a rough correlation network is crucial for the GRN inference. Through analyzing the topology of GRN, it is apparent that GRN is a generally sparse network [29], as shown in Table 1. One significant question is whether the sparsities of the potential network would influence the performance of our framework. As the sparsities of the potential network is controlled by the correlation coefficient threshold, so we investigate how differently GRINCD performs on the DREAM5 datasets under different thresholds.

**FIGURE 4:**
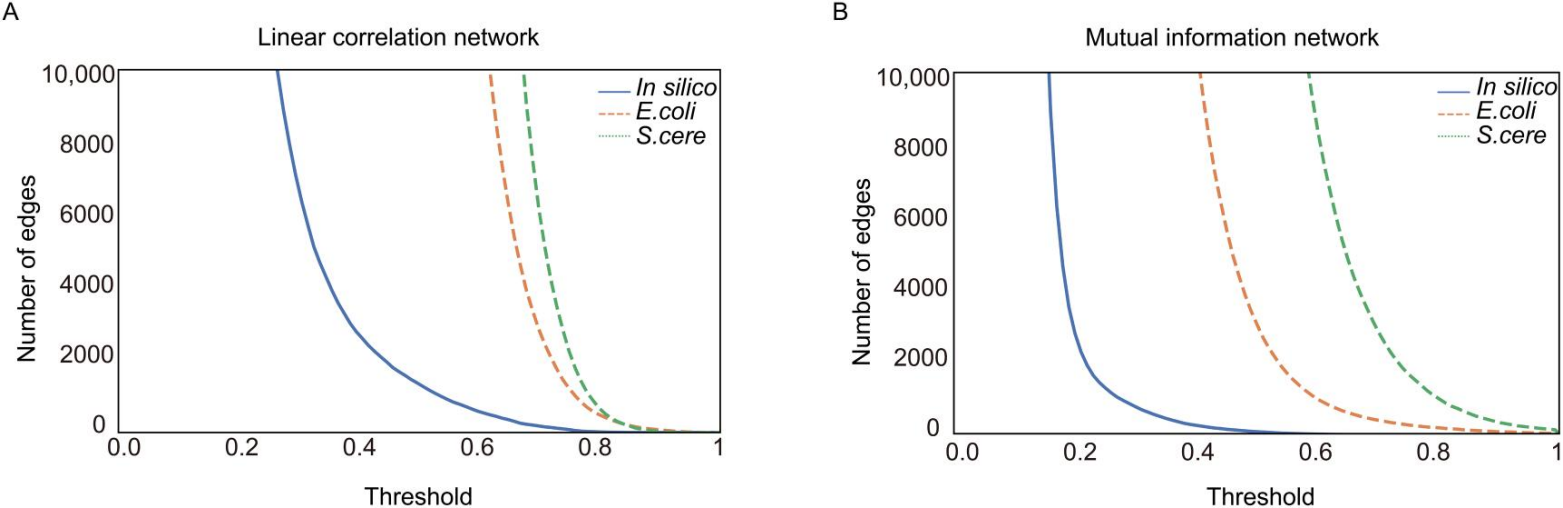
The trends of the number of edges in potential networks with various correlation coefficient thresholds (The ordinates are truncated to 0–10,000). (A) The trend of the number of edges in the linear correlation network. (B) The trend of the number of edges in the mutual information network.

**FIGURE 5:**
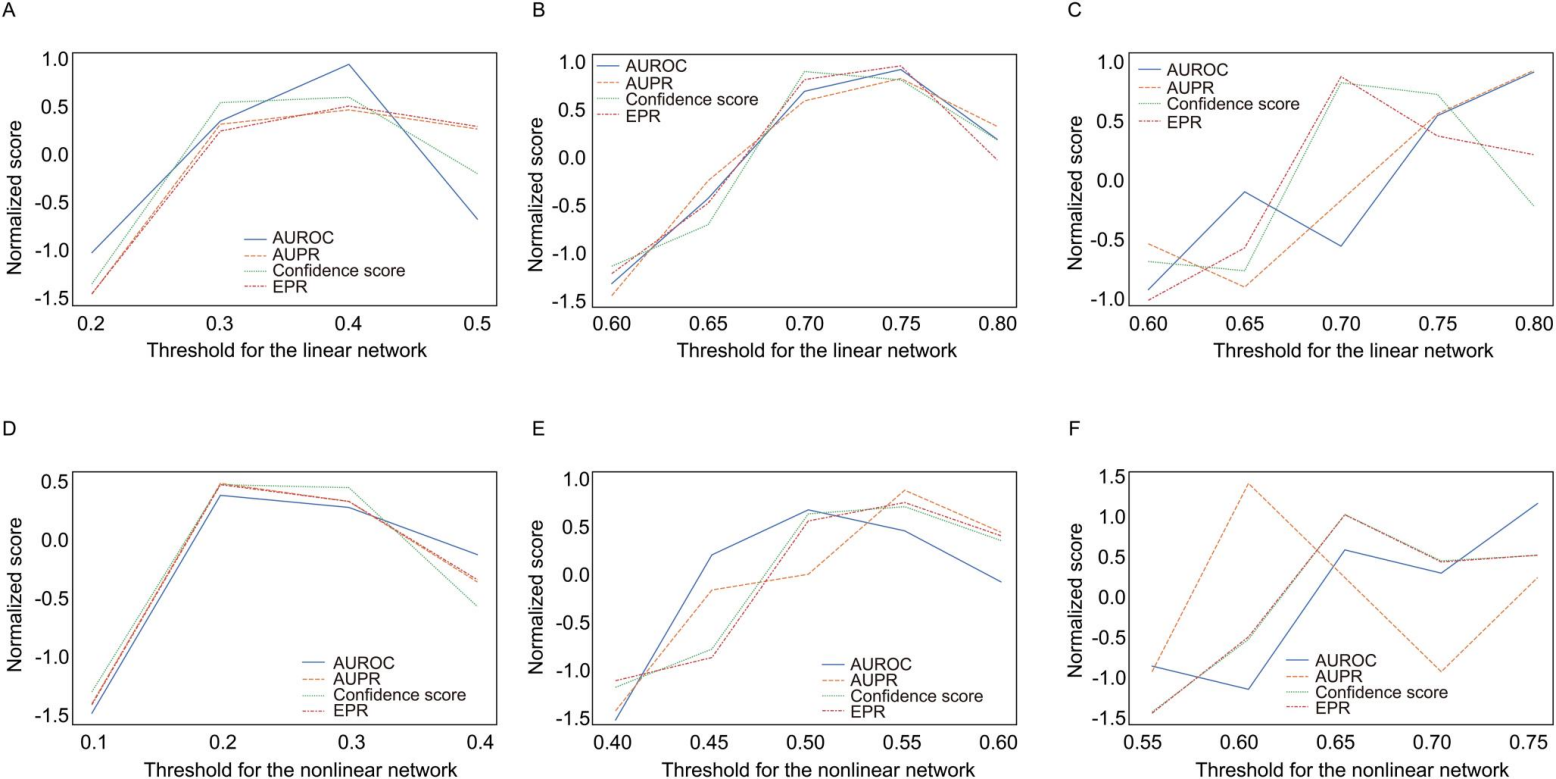
The trend of GRINCD performances with changing thresholds on the DREAM5 data. (A) For in silico, the fixed threshold for nonlinear network is 0.15. (B) For *E.coli*, the fixed threshold for nonlinear network is 0.50. (C) For *S.cere*, the fixed threshold for nonlinear network is 0.65. (D) For in silico, the fixed threshold for linear network is 0.35. (E) For *E.coli*, the fixed threshold for linear network is 0.70. (F) For *S.cere*, the fixed threshold for linear network is 0.70.

As the thresholds increase, the number of edges shows an exponentially decreasing trend for linear correlation‐based networks and a z‐like decreasing trend for mutual information‐based networks (Figure 4 and Figure S8). To simplify the calculation and analyze the general trends, we only capture some deterministic thresholds corresponding to networks with different degrees of sparsities for analyses. As there are two branches with two thresholds, we perform the parameter analyses by fixing one threshold and changing the other one. On the assumption that when the sparsity degree of the co‐expression network is similar to the gold standard, GRINCD has a better performance. We set the fixed correlation cutoff as 0.15, 0.50, and 0.65 for the nonlinear network, and 0.35, 0.70, and 0.70 for the linear network, for in silico, *E.coli*, and *S.cere*, respectively. These thresholds constrain the number of edges in networks to be close to the corresponding gold standard. After fixing the parameters of a network, we analyze the trends under AUROC, AUPR, confidence score, and EPR metrics by setting the other network’s construction threshold. For example, when fixing the threshold of the nonlinear network as 0.50, we observe that four metrics increase with the growing threshold for the linear network, then begin to decline after reaching a peak (Figure 5B). As these four metrics are of different orders of magnitude, we standardize their values before trend analysis. As shown in Figure 5, the result indicates that our framework performs best when the sparsities of potential networks are similar to the gold standards. Moreover, the proposed directed network inference method performs worse when the potential networks become denser.

### 2.6 Combination of gene representation modules and causal modules

To demonstrate the effectiveness of the framework that integrates node embedding and pairwise causal discovery, we combine different gene representation modules and causal discovery modules to infer GRN on DREAM5 datasets. The gene representation modules are GraphSAGE, GCN, and GAT [30], and the causal discovery modules are ANM, IGCI [31], and RECI [32], respectively. The final comparison results are shown in Tables S17–S20. By comparison, we find that the prediction results between different combinations do not show relatively large gaps. For example, for in silico data, the standard deviation among various combinations is 0.027 for AUROCs, 0.010 for AUPRs, 1.373 for confidence scores, and 1.542 for EPRs.

### 2.7 Identification of driver transcription factors for disease development using GRINCD

The transformation from chronic inflammation to cancer has always been a great concern and understanding the transformation mechanism to cancer initiation is beneficial for making early intervention. Existing studies have demonstrated that the regulatory mechanisms indeed change during the state transition of disease progression [33]. Herein, taking colon disease as an example, we apply GRINCD to identify the main differential regulatory relations of normal tissue, inflammatory bowel diseases (IBD), adenoma, and colorectal carcinomas (CRC). We collect the raw gene expression data GSE4183 from gene expression omnibus (GEO) database [34, 35, 36], which contains 53 samples, including 8 normal samples, 15 IBD samples, 15 adenoma samples, and 15 CRC samples. After data preprocessing, 21,653 genes are obtained.

We first remove genes that are certainly not expressed in the colon by excluding all the tissue‐specific genes of other tissues except the colon. Through differential expression analysis (*t*‐test with *p* < 0.01) between normal and CRC stages, we identify 425 differentially expressed genes including 85 TFs and 340 TGs. Tissue‐specific genes and TFs of human cells are collected from TissGDB [37] and TRRUST [38] databases, respectively. We separately apply GRINCD to infer the GRN of each stage.

**FIGURE 6:**
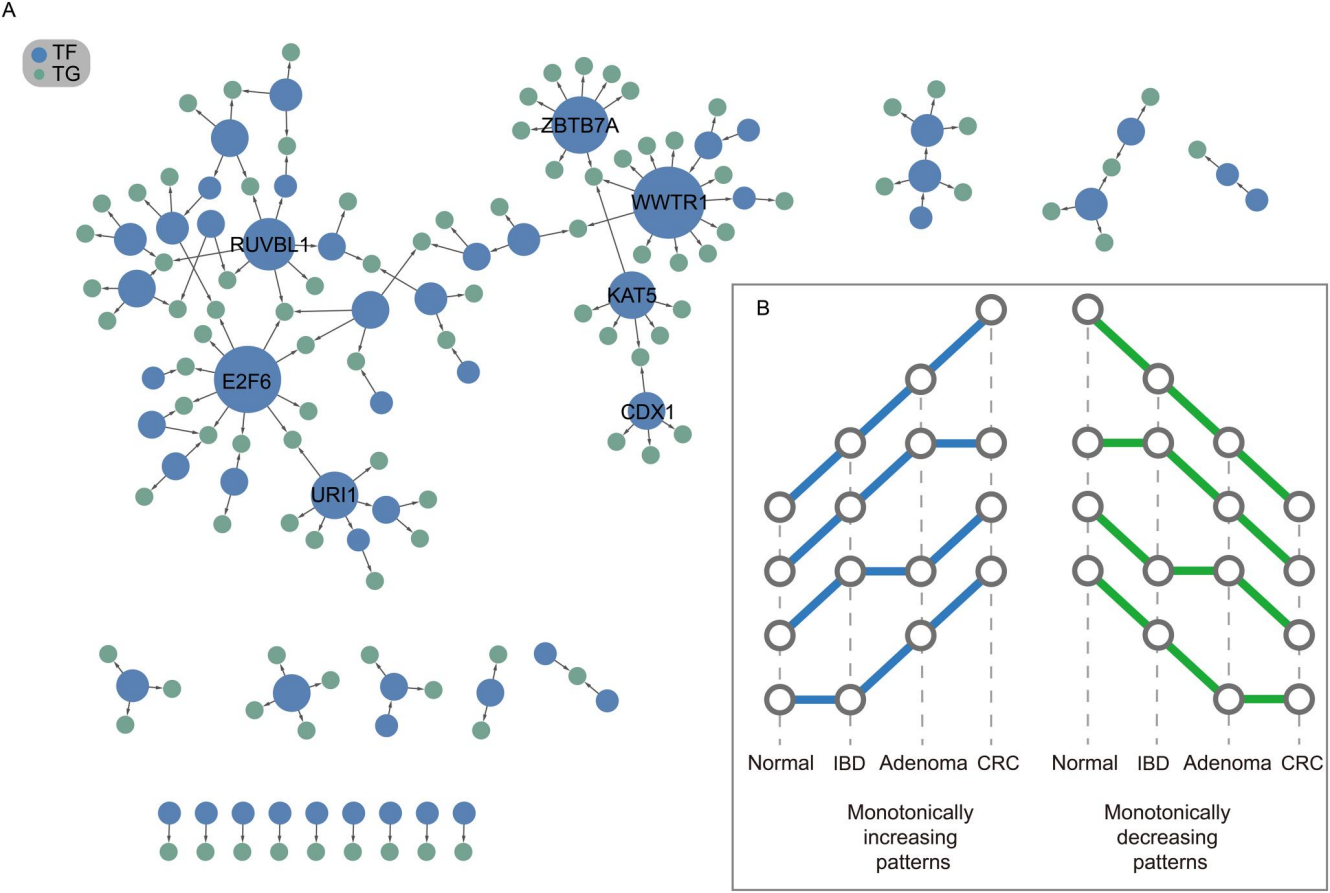
Identification of the key factors driving colon cancer. (A) Monotonically varying regulatory relations; larger size indicates greater importance in cancer development. (B) Definition of monotonically varying; blue lines mean monotonically increasing, and green lines mean monotonically decreasing.

For each regulatory relationship, we analyze the monotonicity of regulation strength in the four stages. We assume that a monotonically increasing or monotonically decreasing regulatory relationship plays a key role in the development of colon cancer (Figure 6B). Setting the threshold of monotonicity judgment to 0.15, we identify 95 monotonically increasing regulatory relations and 45 monotonically decreasing regulatory relations (Figure 6A). Among all the significantly changed regulatory relationships, we observe that some of them share the same TFs and speculate that TFs participating more in disease development‐related regulatory relationships play key roles in cancer initiation. Through our analysis, we suggest WWTR1, E2F6, ZBTB7A, RUVBL1, and URI1 are the potential driver regulators that promote the transformation of colon inflammation to colon cancer. For example, Liu et al. [39] discover that high expression of WWTR1 in colon cancer is related to clinical stage, pathological T‐stage, and lymphatic metastasis. Wang et al. [40] prove that in one or more of the datasets, the mRNA expression level of E2F6 is significantly upregulated in human colorectal cancer (CRC) patients. ZBTB7A acts as an oncogene in CRC patients and can be used as a potential prognostic biomarker and therapeutic target in CRC patients [41]. RUVBL1, as a protein‐coding gene, is associated with diseases including colorectal cancer [42]. Lipinski et al. [43] report that CRC cell lines demonstrate differential dependency on URI1, suggesting that this differential vulnerability of CRC cells is directly linked to URI1C chaperone function. In addition, we conduct the same analysis using two state‐of‐the‐art methods (GINIE3 and TIGRESS). These methods identify two same key factors as identified by GRINCD: WWTR1 and ZBTB7A. It means that GRINCD can not only identify recognized key factors but also discover new key factors.

## 3 DISCUSSION

GRN inference is extremely helpful to understand cell procedures and molecular interplay that govern cells phenotype. Tremendous efforts have been devoted to inferring GRN from observational gene expression data based on generalized correlation, but not causation even though the regulatory mechanism is intrinsically causal. In this study, we propose a GRN adaptation, GRINCD, from the perspective of causal discovery. In view of the complexity of the regulatory relationship between TFs and TGs, we also make full use of the information aggregation and representation capabilities of graph neural networks. We benchmark GRINCD against 17 methods of various principles on transcriptomic data of model organisms, mouse single‐cells, and human single‐cells. Evaluated under different metrics, GRINCD achieves superior or comparable performance in predicting the regulatory relationship of not only TF‐TG but also TF‐TF where generalized correlation‐based methods are unattainable.

Due to the complicated many‐to‐many regulatory relationship, that is, one gene is regulated by multiple TFs and 1 TF can also regulate multiple genes, even other TFs, it is improper to capture and predict the relationship between TF and TG through the expression of a single TF and TG. Hence, we propose to apply GNN to learn node representation at the system level by taking link prediction as the downstream task. Given that the relationship between TF and TG could be linear or non‐linear, we construct two primitive graphs accordingly. On the other hand, the graphs are built by setting an empirically high threshold to improve the credibility of the association and ensure the sparsity of the graph and take link prediction as the downstream task for learning node representation at the system level. Instead of generating a network containing all the observational variables, GRINCD aims to obtain a ranked gene pairs list according to the corresponding causation possibility. Hence, GRINCD can also be used to complete the existing directed biological regulatory network and is not limited to Homo species.

For a certain TF‐TG or TF‐TF to be tested, there may or may not be a connection between them in the original candidate correlation graph. One might wonder whether in the former case, information passed between them could lead to a spurious strong correlation. Since each node usually connects to more than one point, the extra influence of two connected nodes on each other may be counteracted to some extent. In the latter case, we don’t need to worry about this issue. However, in this study, we must admit that data distribution analysis after GNN representation and the relevant theoretical proof is limited, and we will continue to discuss it in our future work.

Time‐series information is usually regarded as playing an important role in causal discovery, that is the cause happens before the result, and the change of the cause occurs first and then leads to the change of the result, but not vice versa. GC provides a typical method. In the GRN inference scenario, GC aims to infer regulation on the notion that changes in regulators should precede changes in their targets in time. However, there are few available biometric data with real‐time tags and appropriate time intervals, which limits the practical application of GC‐based methods. We take Scribe, which introduces GC to the GRN inference, for comparison under various evaluation indexes. However, as indicated in Figures S1 and S2, the performance of Scribe on scRNA‐seq data with pseudo‐time information does not perform significantly better than other methods. In all evaluation results, Scribe outperforms 25 (20.83%) results, and the largest difference is 0.049 for AUROC; outperforms 10 (8.33%) results, and the largest difference is 0.02 for AUPR; it outperforms 17 (14.17%) results, and the largest difference is 17.87 for confidence score; it outperforms 20 (16.67%) results and the largest difference is 0.783 for EPR. Altogether, the performance of Scribe is even below the average. Regarding this, we speculate that the pseudo‐time of a single‐cell involves irregular time intervals, and such behavior is unfavorable for time‐series data analyses and predictions.

Besides, application of GRINCD to infer various and crucial regulatory relationships during the transition from inflammatory bowel disease to colorectal cancer (COAD) reveals that a certain bunch of TFs are the driving force of cancer development. GRINCD fills the gap of effective causal‐based GRN inference on large‐scale datasets and leads causal discovery to a broader biological application. GRINCD is also expected to be used in cell differentiation, cell state transition, cell type determination, and other studies.

In conclusion, in this study, the proposed framework of GRN inference, GRINCD, achieves good performance on multiple datasets and explores potential key factors affecting cancer development through multi‐stage GRN construction. GRINCD studies the efficient application of causal discovery algorithms on large‐scale datasets. It may have other potential applications, such as in molecular regulation at the multi‐omics level by applying a heterogeneous network to investigate the regulatory mechanism.

## 4 MATERIALS AND METHODS

We define the GRN inference task as follows: GRN is a weighted directed bipartite graph <*F*,*G*,*E*,*W*>, where *F* is the set of TFs, *G* is the set of TGs. *E* is the set of directed edges including all possible <TF, TF> and <TF, TG> pairs; note that neither <TG, TG> pair belongs to *E* in this study. *W* is a |TF*s*| × |TF*s* + TG*s*| regulatory confidence matrix, *W*_*i*,*j*_ corresponds to the confidence of the edge <TF_*i*_, TF_*j*_> when *i* > *j* or < TF_*i*_, TG_*j*‐|*TFs*|_> when *i* < *j*. We set *W*_*i*,*i*_ = 0 under the assumption that there exists no self‐regulation in GRN. *M* represents a *m* × *n* gene expression matrix with rows as *m* samples, columns as *n* genes and *n* equals |TF*s* + TG*s*|. Our goal is to calculate *W* by taking *F*, *G*, and *M* as inputs.

### 4.1 Datasets and pre‐processing

We apply our framework to 15 datasets to validate the performance. First, we adopt three benchmark datasets in silico, *E.coli*, and *S.cere* from the DREAM5 challenge [1]. In silico is a synthetic dataset whose expression matrix is generated by the simulated software GeneNetWaver (GNW) [44]. GNW regards GRN as a stochastic dynamical system and utilizes ODE [45, 46] to model GRN. Gene expression of *E.coli* and *S.cere* are obtained from GEO database. The corresponding three gold standards of GRN are: (i) the known network for the in silico dataset, (ii) experimentally validated interactions from a curated database RegulonDB [47] for *E.coli*, and (iii) a high‐confidence set of interactions supported by genome‐wide TF binding data [48], together with evolutionarily conserved binding motifs [49] for *S.cere*. We also evaluate the inference performance of GRINCD at the single‐cell level; two additional scRNA‐seq datasets GSE75748 and GSE48968 downloaded from the GEO database are used, which are hESC and mDC, respectively.

We follow the BEELINE framework [50], a platform for benchmarking GRN inference, which uses principal component analysis and generalized additive model to process the raw scRNA‐seq data. In addition, there are three kinds of gold standards for each scRNA‐seq dataset according to the information sources: cell‐type‐specific ChIP‐seq from ChIP‐Atlas [51], non‐specific ChIP‐seq from TRRUST [38], and functional interaction networks collected from the STRING database [52]. For each of these two scRNA‐seq datasets, hESC and mDC, we take significantly varied TFs, and the top 500 and 1000 most‐varying TGs into consideration [50], respectively. Finally, taking the combination, we adopt 15 benchmark datasets. The detailed information on these datasets is listed in Table 1. As for single‐cell datasets, “CTS” means this dataset is generated using the cell type‐specific gold standard, “NS” means this dataset is generated using the non‐specific gold standard, “STR” means this dataset is generated using the STRING gold standard, “500” and “1000” represent different parameters in data preprocessing. For mDC and hESC datasets, there is no intersection between the three different types of gold standards, so the evaluation of method results using different gold standards is relatively more comprehensive.

For the raw gene expression data, expression values often vary among genes. To make our method more general and effective, we normalize *M* using z‐score to get a standardized gene expression matrix *M* ^′^.

(1) $$\begin{array}{c} M_{.j}^{\prime }=\frac{M_{.j}-\bar{M_{.j}}}{\sqrt{\frac{\sum_{i=1}^{m}{\left(M_{i,j}-\bar{M_{.j}}\right)}^{2}}{m}}} \end{array}$$

### 4.2 Constructing potential networks

In order to guide gene representation learning, we first construct an initial co‐expressed network in which the edges represent correlations between genes. Pearson correlation and mutual information are used to measure the linear and nonlinear relationships between genes, respectively. Let *R* be the relevant correlation coefficients matrix and *N* be the adjacent matrix of co‐expressed network generated after setting a proper threshold.

(2) $$\begin{array}{c} N_{i,j}=\left\{\begin{array}{c} 1,R_{i,j}\geq \text{threshold}\;\text{and}\;i\neq j\\ 0,R_{i,j}<\text{threshold}\;\text{or}\;i=j \end{array}\right. \end{array}$$

### 4.3 Learning gene representation with GraphSAGE

Identification of a causal regulatory relation between genes that are in a system is non‐trivial. Directly using pairwise gene expression vectors in *M* ^′^ would omit the effect of other possible regulatory factors on two genes. In order to address this issue, we aggregate useful information about each gene from a co‐expressed network. With a co‐expressed network *N* and the gene expression matrix *M* ^′^ as input, we integrate node‐embedding with a supervised link classification task using a two‐layer GraphSAGE and a binary classifier.

As shown in Figure 1B, we first conduct uniform random walks over the whole co‐expressed network, *N*, and extract positive links from those walks. Negative links are sampled randomly from the degree distribution of the network, *N*. We generate an equal number of positive links and negative links considering the balance of the samples. Afterward, with sampled links and their corresponding labels as input, the objective function is as follows:

(3) $$\begin{array}{c} CorssEntropy=-\sum_{k}\left({label}_{k}*\log\left({prob}_{k}\right)+\left(1-{label}_{k}\right)*\log\left(1-{prob}_{k}\right)\right) \end{array}$$

where *label*_*k*_ is the label of edge *k*, *prob*_*k*_ is the probability that edge *k* is predicted to be positive. Adma [53] is used to optimize the network.

Once the whole model is well trained, a two‐layer GraphSAGE could generate a gene representation matrix $\tilde{M}$. In detail, the layer of GraphSAGE induces the structure of a co‐expressed network, and updates gene representation according to the obtained network knowledge. GraphSAGE follows the procedure of neighbor node sampling and information aggregation. As for sampling, we assign the fixed sampling size and hops for each node to facilitate feeding the neural network (NN) with bath data of the same size. Bootstrap is conducted if the number of candidate neighbors is less than the sampling size. After that, we get a neighbor node set *NS*(*g*) for node *g* and use the node information in *NS*(*g*) to update the representation of node *g* by information aggregation. In mathematical language, information aggregation of node *g* in the *lth* layer GraphSAGE is formulated as follows:

(4) $$\begin{array}{c} h_{g}^{l}=\sigma \left(\Theta \cdot \text{concat}\left(h_{g}^{l-1},\text{mean}\left(h_{u}^{l-1},\forall u\in NS\left(g\right)\right)\right)\right) \end{array}$$

where *h* is the feature vector of node *g*, *Θ* is the weight matrix to optimize, *σ* is the activation function.

### 4.4 Identifying the causal regulatory relationship

After the representation learning, we get the new embedding of gene expression $\tilde{M}$ which aggregates some of its correlated genes’ information to itself. Pairwise causal discovery methods usually identify causal direction between two genes by discovering asymmetry between specific distributions. Here, we apply ANM, a model that uses nonlinear relationships and Gaussian noise to identify causal directions, to judge the causal direction between two genes.

**FIGURE 7:**
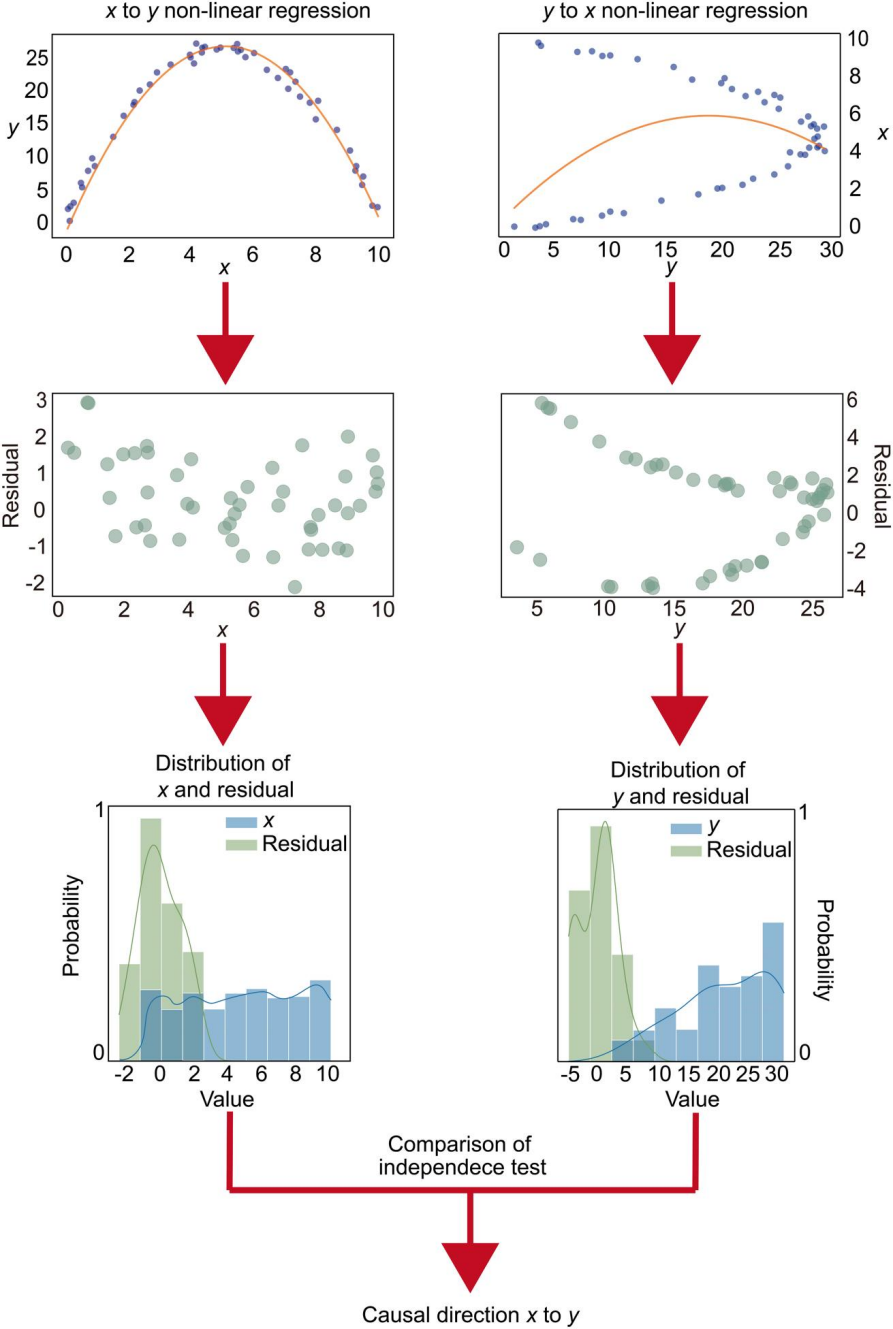
Inference of causal direction between variable *x* and *y* via additive noise model (ANM). We randomly generated 50 points, where the relationship between vector *x* and vector *y* satisfies: *y* = −*x* ^2^ + 10*x* + 3. Then Gaussian noise is also added to *y*. ANM conducts two nonlinear regressions, *x* to *y* and *y* to *x*. Then it performs two independence tests between the regression residual and independent variable. Finally, it takes the more significant one as the causal direction.

ANM assumes a nonlinear relationship between the cause and outcome variables and an independent additive Gaussian noise. A nonlinear relationship conforms to the complex GRN regulation, and the introduction of Gaussian noise into the model is also a common way to simulate complex perturbations. We implement ANM by conducting two regressions in different directions to get the regression residuals, and then identify the causal direction by verifying the independence between the residuals and the independent variables. For example, given two gene expression vectors *x* and *y*, we can infer the causal direction between them via ANM (Figure 7).

According to the ANM, the distribution of a variable *x* is generated as follows:

(5) $$\begin{array}{c} x=f\left(x_{pa}\right)+\delta \end{array}$$

where *x*_*pa*_ denotes causal variable of *x*, *f* is an arbitrary function (and different variables may correspond to different *f)*, and *δ* is an independent noise. According to the different forms of function and different distributions of noise, the above models can be divided into the linear Gaussian model, the nonlinear Gaussian model, the linear non‐Gaussian model, and the nonlinear non‐Gaussian model. Linear non‐Gaussian acyclic model [54] and PNL are two widely used models.

Concerning the simplest case of two variables *x* and *y*, the causal direction between them can be identified by the non‐linear Gaussian model. The identification of causal direction between two variables is based on the assumption that the cause and noise are independent of each other. We estimate noise by Gaussian process regression (GPR) [55], which is a classic regression method that could satisfy the non‐linear relation between variables and Gaussian noise. GPR is a non‐parametric, Bayesian approach to regression and works well on small datasets, and has the ability to provide uncertainty measurements on the predictions. Residuals from GPR results are regarded as noise; we validate the independence between noise and causal variable by the hilbert‐schmidt independence criterion (HSIC) [56]. HSIC is a kernel‐based independence criterion whose main purpose is to measure a distributional difference between two variables. The principle of HSIC is to define the hilbert‐schmidt cross‐covariance operator on the reproducing kernel hilbert space and then derive a statistic magnitude for measuring independence. The test statistics of HSIC are used as the measurement of causal direction as follows:

(6) $$\begin{array}{c} \text{ANM}\left(x,y\right)=\text{HSIC}\left(\;\left(\text{GPR}\left(\;x,y\;\right)-x\;\right),x\right) \end{array}$$

Instead of just using one direction as our result, we define a directional propensity score *W* which measures the difference in scores between the two directions as follows:

(7) $$\begin{array}{c} W_{i,j}=\left\{\begin{array}{ll} \text{ANM}\left({\tilde{M}}_{\cdot i},{\tilde{M}}_{\cdot j}\right)-\text{ANM}\left({\tilde{M}}_{\cdot j},{\tilde{M}}_{\cdot i}\right), & i\neq j\\ 0, & i=j \end{array}\right. \end{array}$$

In addition to GPR, the NN is also a powerful nonlinear regression model. In order to compare the impact of using GPR and a NN to implement ANM on the final result, we also use a 2‐layer fully connected NN with sigmoid as the activation function to implement ANM and compare the final results on the DREAM5 datasets with the original GRINCD. It shows that using a NN to implement ANM will not greatly improve the results (Tables S21–S24). On the contrary, the training of a NN is more time‐consuming and resource‐consuming, so using GPR to implement ANM is a better choice.

### 4.5 Ensemble results from linear and nonlinear pipelines

So far, we get two confidence matrices *W*_*li*_ and *W*_*non*‐*li*_ respectively from the linear and nonlinear pipelines. The synthesis of these two pipelines could lead to more robust results. Here, we use Borda count voting [57] with a weight *λ*, a simple but effective method for synthesis as follows. In this work, we set the parameter *λ* to 0.5.

(8) $$\begin{array}{c} W=\lambda W_{li}+\left(1-\lambda \right)W_{non-li},\lambda \in \left(0,1\right) \end{array}$$

### 4.6 Evaluation metrics

Evaluating the performance of methods for GRN inference is non‐trivial. Different models often use different evaluation methods and metrics. Here, we treat the evaluation of GRN inference as a binary classification task.

We adopt the commonly used metrics AUROC and AUPR to assess the performance of GRN inference methods. AUROC and AUPR are calculated as follows: (i) setting a threshold for the output ranked list, we calculate the corresponding true‐positive rate (TPR), FPR, recall, and precision, respectively; (ii) we continuously adjust the threshold to obtain a series of the above four values; (iii) these values are then used to plot the receiver operating characteristic (ROC curves and precision‐recall (PR) curves; (iv) the area under the ROC curve (AUROC) and the area under the PR curve (AUPR) are calculated at last.

In practice, we calculate TPR, FPR, recall, and precision in different ways according to the length of the output list. When the length of the output list is equal to the number of all possible gene pairs, those four values are computed as usual. Otherwise, they are computed using sampling and filling methods [58]. Mathematically, they are defined as:

(9) $$\begin{array}{c} \text{Recall}=\text{TPR}=\left\{\begin{array}{c} \frac{\text{TP}}{P},\;k\leq L\\ \frac{{\text{TP}}_{L}+∆\text{TP}}{P},\;k>L\; \end{array}\right. \end{array}$$

(10) $$\begin{array}{c} P\text{recision}=\left\{\begin{array}{c} \frac{\text{TP}}{k},\;k\leq L\\ \frac{{\text{TP}}_{L}+∆\text{TP}}{k},\;k>L \end{array}\right. \end{array}$$

(11) $$\begin{array}{c} \text{FPR}=\left\{\begin{array}{c} \frac{\text{FP}}{N},\;k\leq L\\ \frac{k-{\text{TP}}_{L}-∆\text{TP}}{N},\;k>L \end{array}\right. \end{array}$$

(12) $$\begin{array}{c} ∆\text{TP}=\frac{\left(k-L\right)*\left(P-{\text{TP}}_{L}\right)}{T-L} \end{array}$$

where TP, FP, TN, and FN are the numbers of true positive, false positive, true negative and false negative respectively, *k* is the number of edges that are judged as positive according to the specified threshold, *L* is the length of the output list, *P* and *N* are respectively the numbers of positive and negative in the gold standard, *T* is the number of all possible gene pairs, TP_*L*_ is the number of true positive in the output list. As for GRINCD, the output list contains all possible gene pairs, so we calculate the above values using the first condition that is, *k*≤ *L*.

Moreover, enlightened by DREAM, besides AUROC and AUPR, we use the confidence score that denotes the difference between the inferred result and a random network corresponding to a null distribution to assess the reliability of the result. We generate 100,000 random networks and evaluate the corresponding results, thus obtaining the discrete distribution of AUROC and AUPR for each dataset. DREAM5 uses stretched exponential functions to fit the discrete distribution to get the approximate probability of any specific AUROC or AUPR value [58]. In order to improve the efficiency and accuracy of fitting, we adopt Gaussian kernel density estimation to fit the distribution. However, in practice, when evaluating two methods using the confidence score, the result may perform a huge gap, for example, the difference value of confidence scores between GINIE3 and BMA [59]. So we limit the confidence score to 100.

We also adopt another metric EPR defined by BEELINE [50]. Foremost, early precision is defined as the number of true positives in the top‐*q* edges, where *q* equals the number of edges in the gold standard. For a specific inference output of a particular dataset, the EPR is computed as the ratio of the output’s early precision value to a random predictor’s early precision.

Although both EPR and the confidence score measure the reliability of a prediction in different ways, the discrepancy in the values from the two specific methods does not absolutely represent the difference in their inference ability. In other words, these two metrics are nonlinear, and we only use them to show an intuitive comparison.

### 4.7 Implementation of GRINCD

In our framework, we try to make full use of hardware resources, including CPU and GPU for parallel processing to reduce time costs. Most scripts are coded with Python, except some source codes of benchmark methods which are directly downloaded from the repository. These codes may be coded with R or Julia. The codes generated during this work are available on GitHub.

## AUTHOR CONTRIBUTIONS

**Ke Feng**: Data curation; methodology; visualization; software; writing – original draft. **Hongyang Jiang**: Writing – review and editing. **Chaoyi Yin**: Writing – review and editing. **Huiyan Sun**: Conceptualization; funding acquisition; supervision; Writing – review and editing.

## CONFLICT OF INTEREST STATEMENT

The authors Ke Feng, Hongyang Jiang, Chaoyi Yin, and Huiyan Sun declare that they have no conflict of interest or financial conflicts to disclose.

## ETHICS STATEMENT

All procedures performed in studies involving animals were in accordance with the ethical standards of the institution or practice at which the studies were conducted, and with the 1964 Helsinki declaration and its later amendments or comparable ethical standards.

## Supporting information

Supplementary Information S1

## Data Availability

The data that support the findings of this study are open source and available from the corresponding website mentioned in the article.
