# Supplementary material for: Gene regulatory network inference based on causal discovery integrating with graph neural network
Source: Quant Biol. 2023 Dec 22;11(4):434–50. doi: 10.1002/qub2.26 (PMC12806863; doi:10.1002/qub2.26)
Supplement: Supplementary file 1 — Supplementary Information S1 [file QUB2-11-434-s001.pdf]

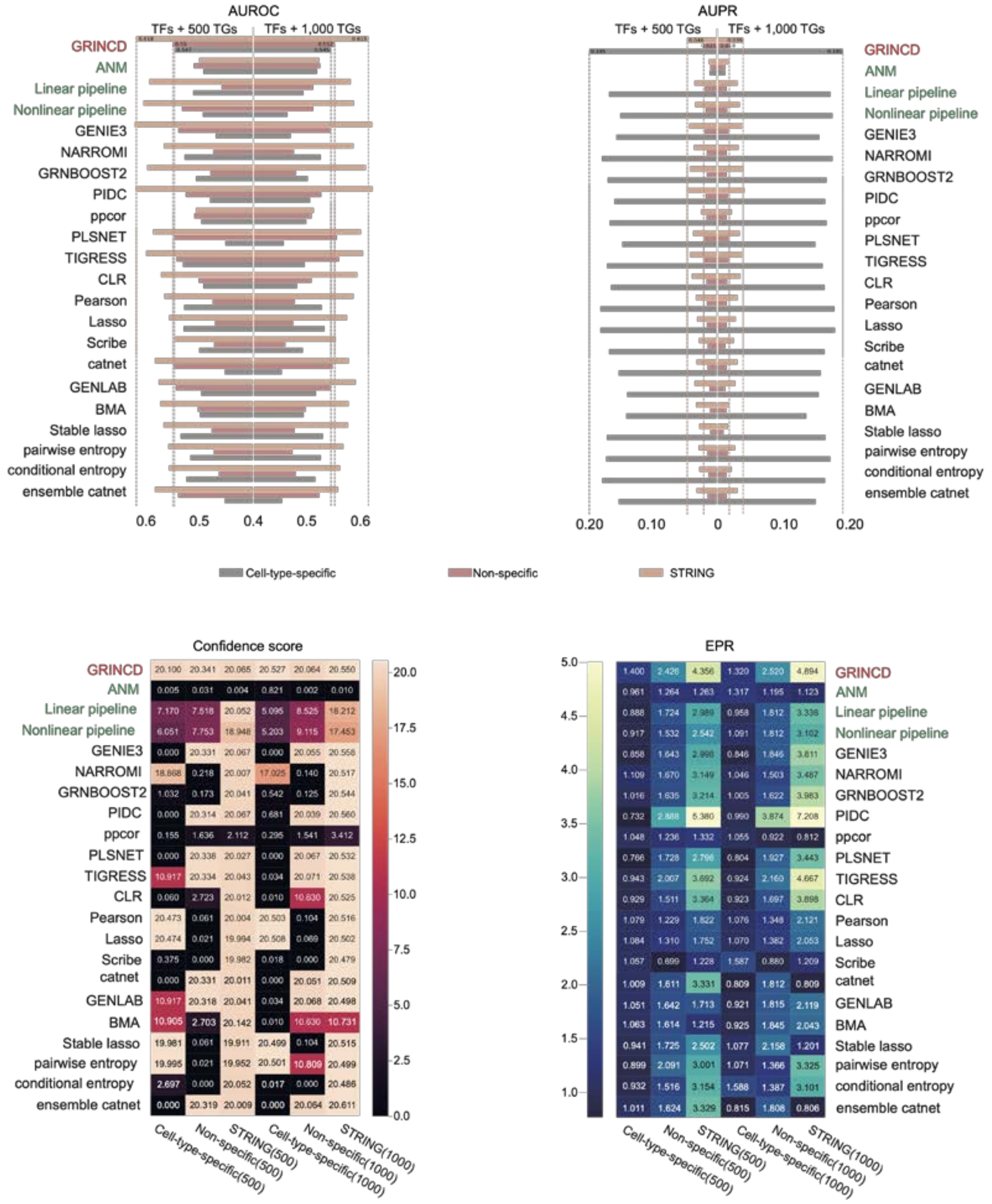

FIGURE S1 Summary of the GRN prediction performance on the hESC datasets under different evaluation metrics. The dotted lines represent the performances of GRINCD, and the methods highlighted in green represent the results of ablation experiments.

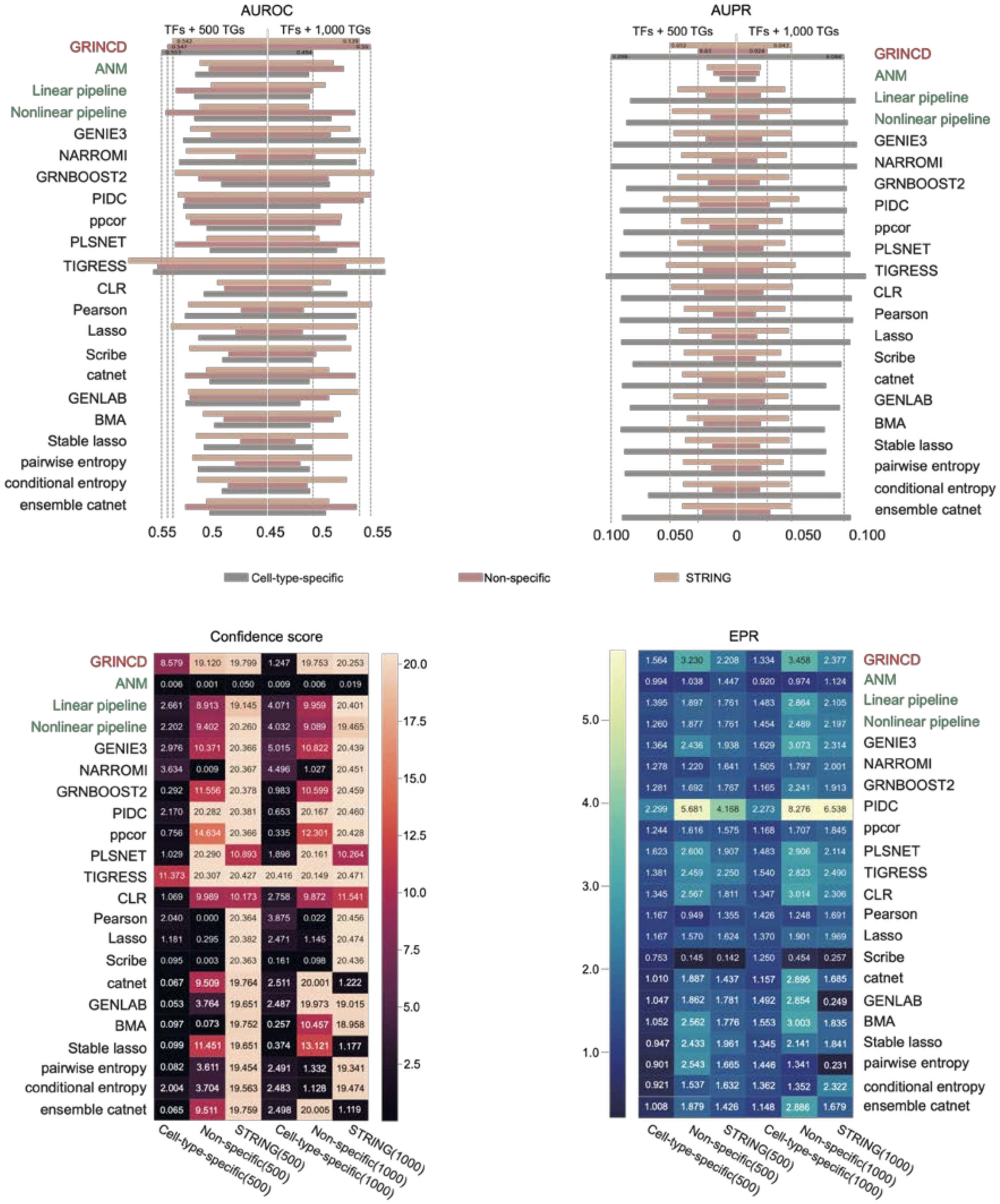

FIGURE S2 Summary of the GRN prediction performance on the mDC datasets under different evaluation metrics. The dotted lines represent the performances of GRINCD, and the methods highlighted in green represent the results of ablation experiments.

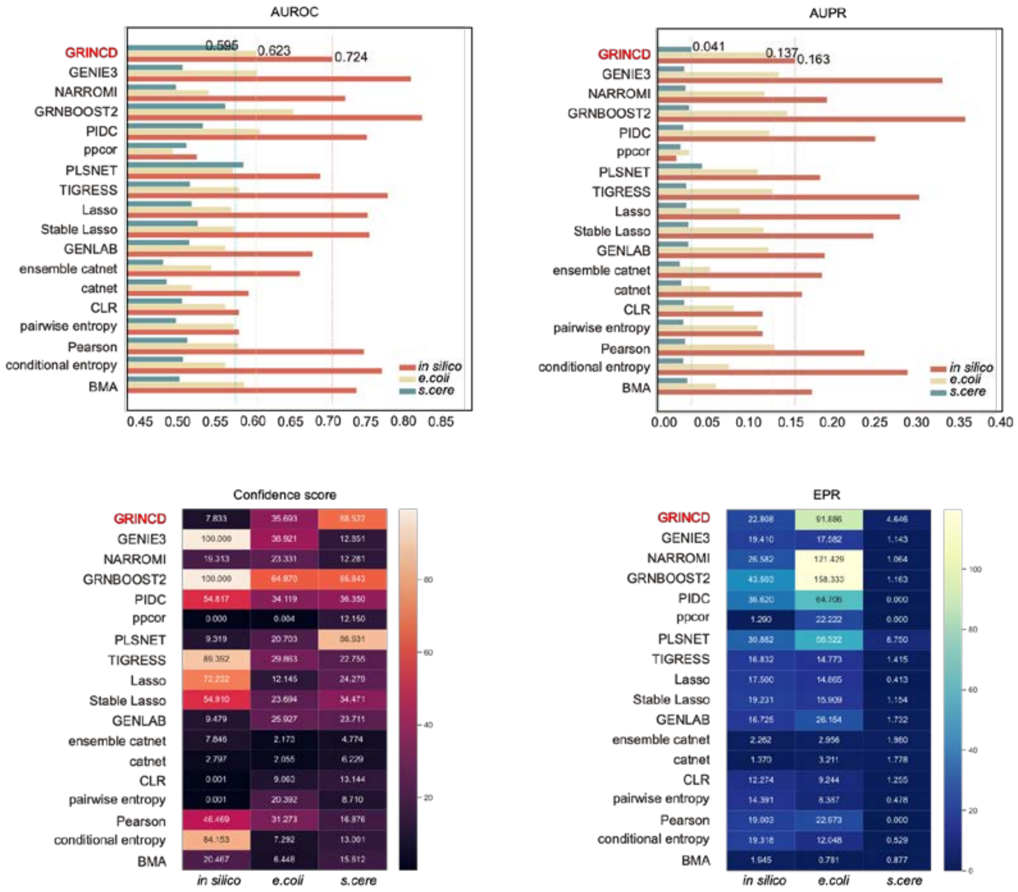

FIGURE S3 Summary of the GRN prediction performance on the DREAM5 datasets in TF-TF evaluation. The dotted lines represent the performances of GRINCD.

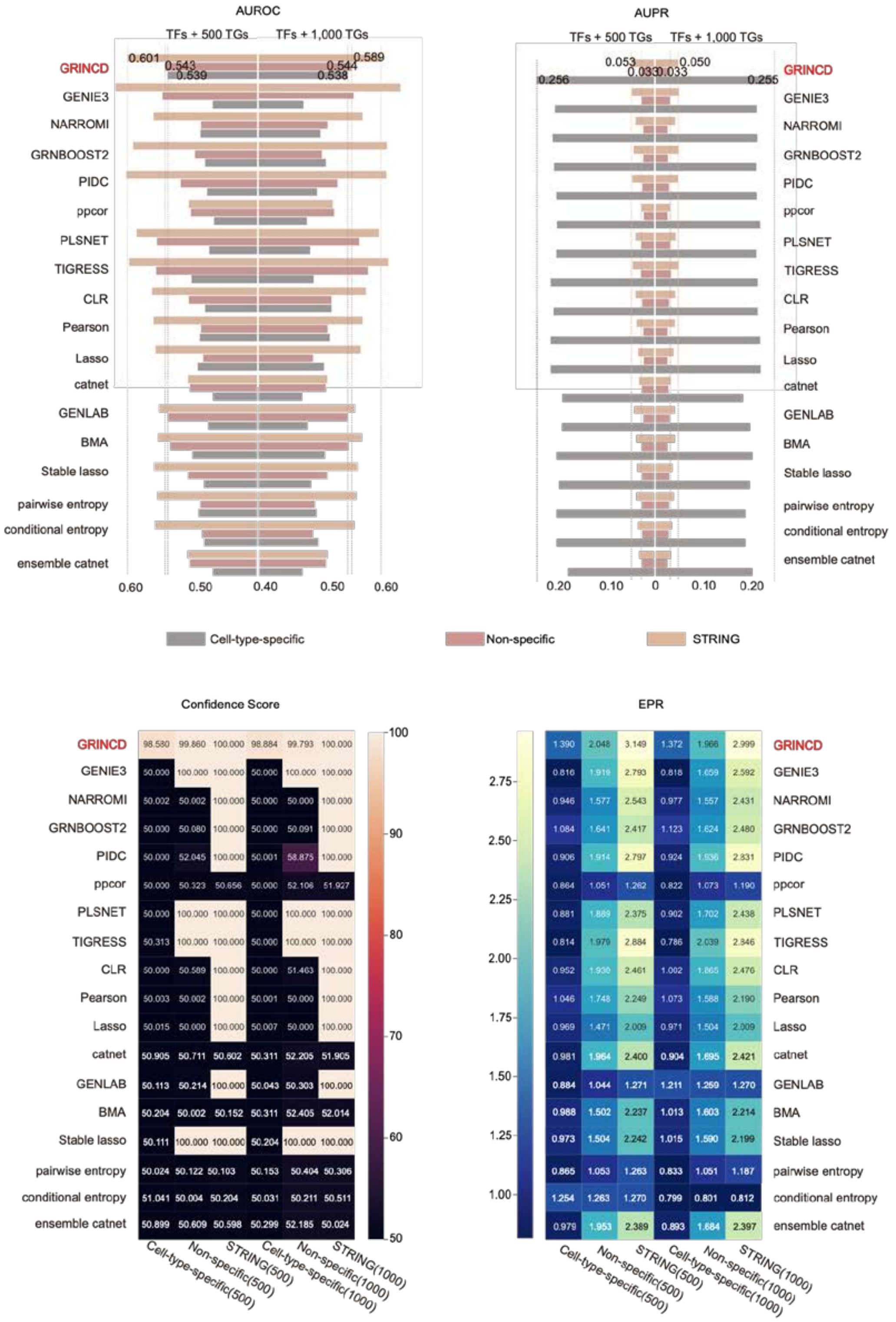

FIGURE S4 Summary of the GRN prediction performance on the hESC datasets in TF-TF evaluation.

The dotted lines represent the performances of GRINCD.

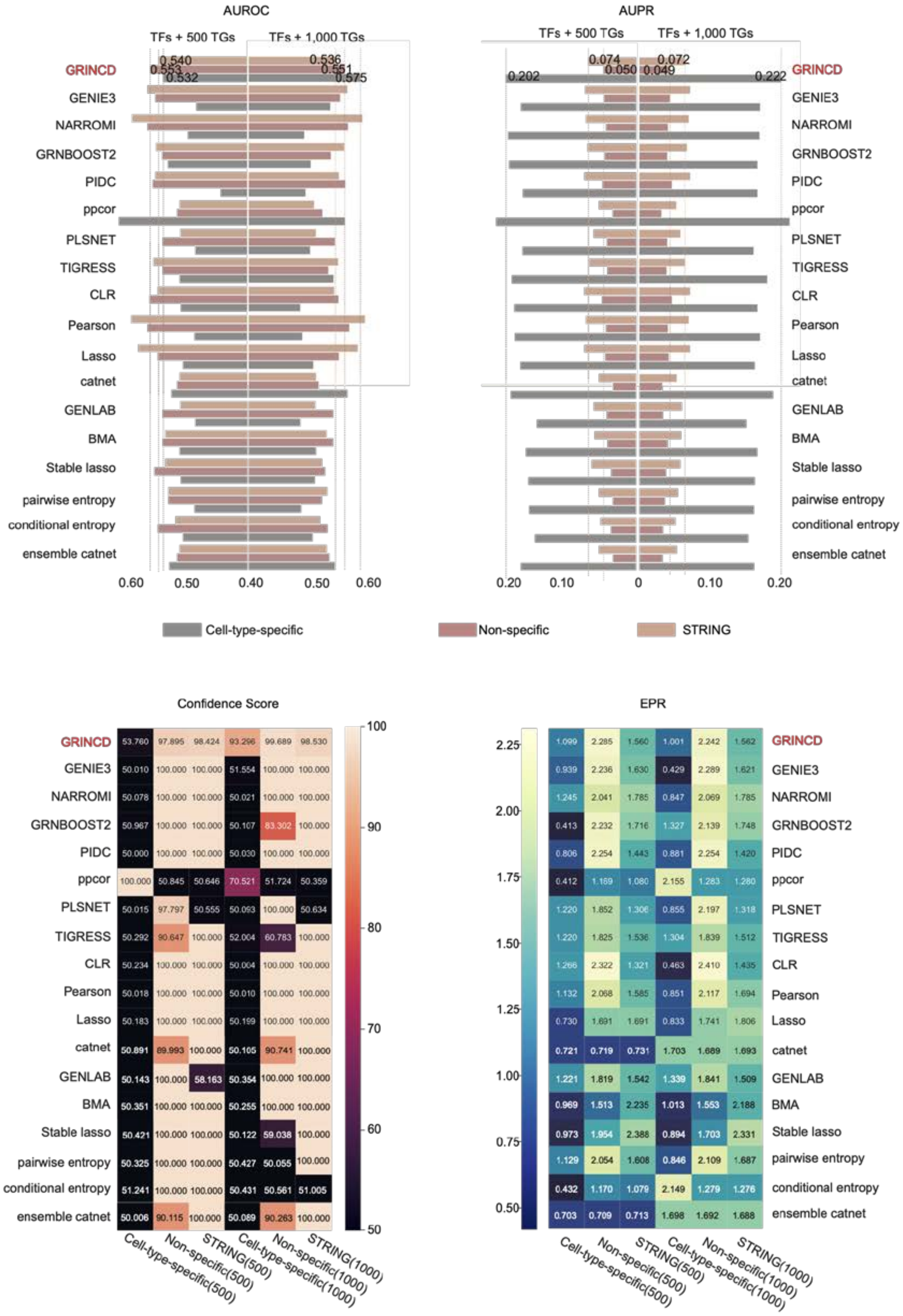

FIGURE S5 Summary of the GRN prediction performance on the mDC datasets in TF-TF evaluation. The dotted lines represent the performances of GRINCD.

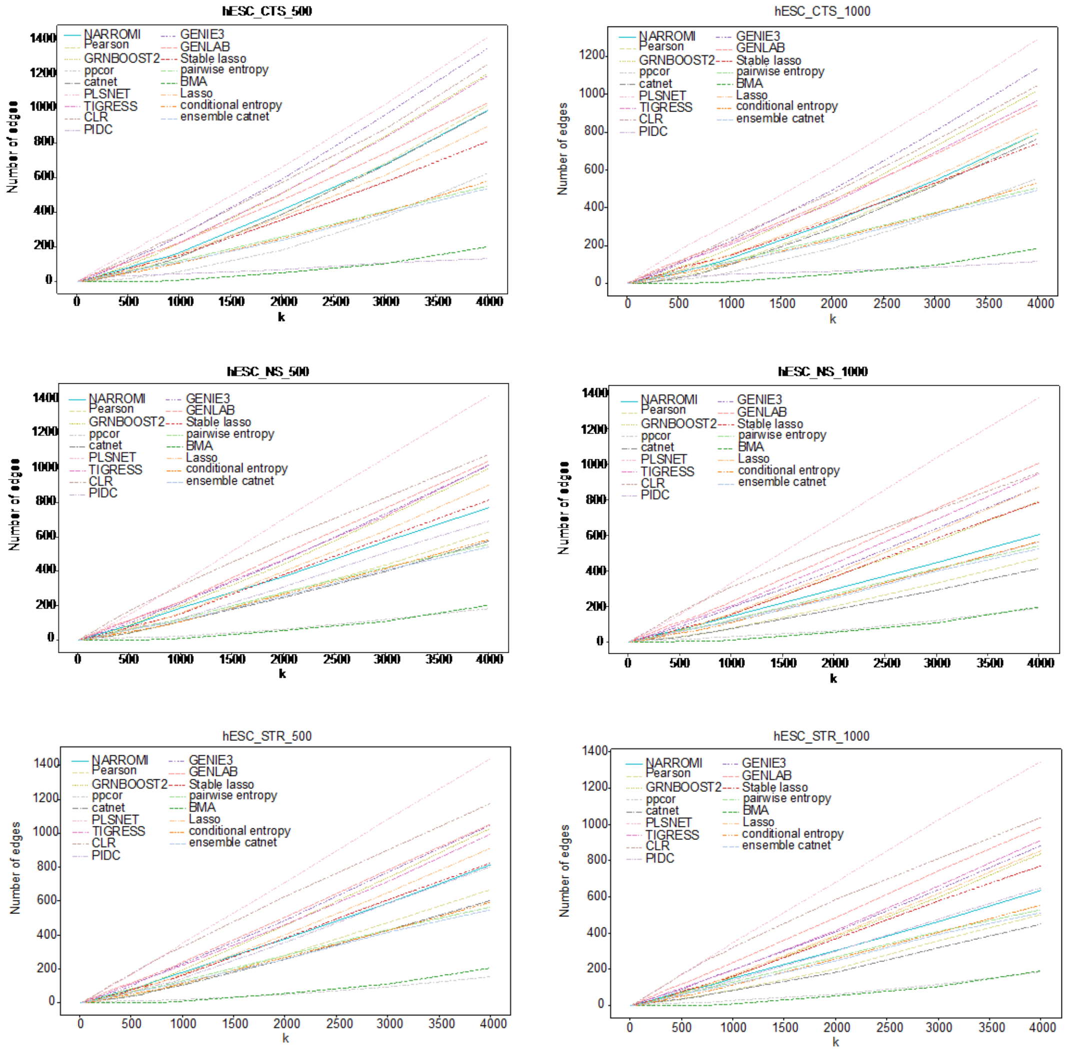

FIGURE S6 The intersection of top-k regulatory relationships between GRINCD and other methods on the hESC datasets.

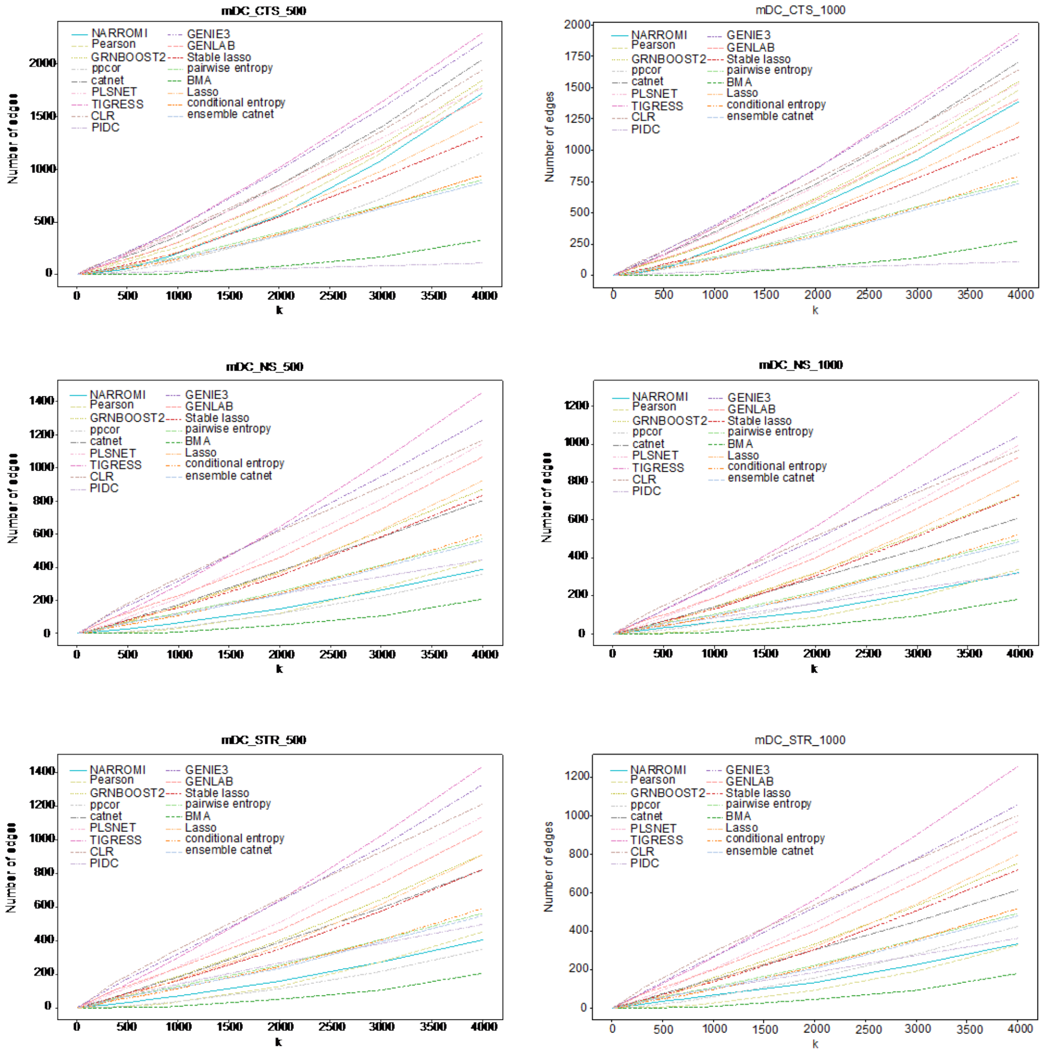

FIGURE S7 The intersection of top-k regulatory relationships between GRINCD and other methods on the mDC datasets.

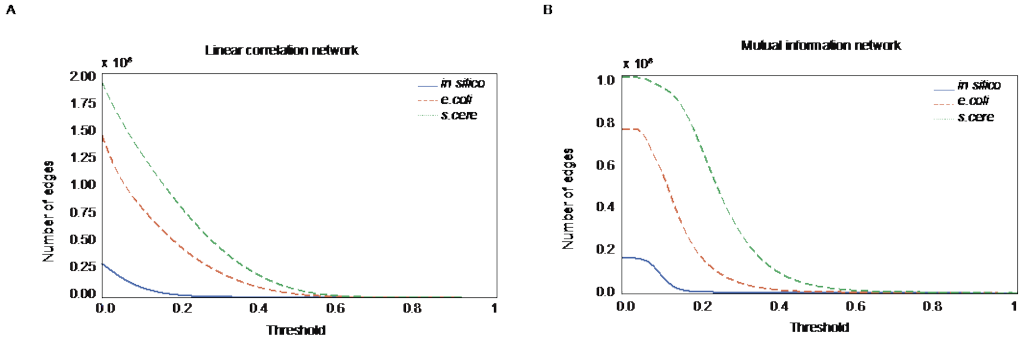

FIGURE S8 The trends of the number of edges in potential networks with various correlation coefficient thresholds. (A) The trend of the number of edges in the Linear Correlation Network. (B) The trend of the number of edges in the Mutual Information Network.

TABLE S1 Results of methods on *s.aureus* dataset.

Methods	AUPR	AUROC	confidence score	EPR
GRINCD	0.071	0.654	25.684	25.884
GENIE3	0.095	0.622	9.810	9.330
TIGRESS	0.087	0.584	0.786	6.480
Lasso	0.098	0.578	1.942	3.548
Stable lasso	0.051	0.508	1.175	0.694
CLR	0.081	0.558	0.829	10.773
pairwise entropy	0.068	0.547	2.340	6.547
conditional entropy	0.058	0.622	0.183	2.544
Pearson	0.090	0.623	0.729	4.273
GENLAB	0.084	0.526	11.333	8.530
ensemble catnet	0.068	0.529	0.010	1.967
BMA	0.062	0.561	6.175	2.438
catnet	0.068	0.530	0.034	3.189
narromi	0.059	0.536	0.505	17.655
GRNBOOST2	0.094	0.620	5.828	20.634
PIDC	0.069	0.563	6.056	22.369
ppcor	0.030	0.484	0.000	5.197
plsnet	0.074	0.557	23.520	23.753

TABLE S2 Accuracy rate of top-k regulation relationship of methods on *in silico* dataset.

Methods	k=50	k=100	k=200	k=500	k=1000	k=2000	k=3000	k=4000
GRINCD	0.901	0.825	0.724	0.602	0.484	0.348	0.273	0.230
catnet	0.840	0.780	0.775	0.686	0.506	0.451	0.355	0.290
GENLAB	0.880	0.830	0.770	0.680	0.559	0.389	0.306	0.251
NARROMI	0.620	0.640	0.635	0.556	0.437	0.325	0.274	0.243
Pearson	0.640	0.720	0.670	0.596	0.493	0.386	0.315	0.272
Stable lasso	0.800	0.770	0.640	0.652	0.565	0.459	0.373	0.312
GRNBOOST2	0.960	**0.960**	0.930	0.858	0.745	**0.560**	**0.445**	**0.374**
pairwise entropy	0.780	0.800	0.715	0.592	0.490	0.390	0.330	0.282
ppcor	0.100	0.070	0.050	0.038	0.035	0.033	0.029	0.027
BMA	0.160	0.140	0.125	0.142	0.141	0.157	0.160	0.187
PLSNET	0.920	0.870	0.755	0.622	0.482	0.358	0.290	0.243
TIGRESS	0.980	**0.960**	**0.945**	**0.874**	0.725	0.520	0.402	0.333
CLR	0.920	0.940	0.890	0.774	0.637	0.462	0.367	0.304
PIDC	0.880	0.940	0.865	0.734	0.600	0.434	0.350	0.291
GENIE3	**1.000**	0.950	0.870	0.788	0.689	0.508	0.395	0.338
Lasso	0.860	0.830	0.805	0.798	**0.746**	0.528	0.408	0.334
conditional entropy	0.400	0.430	0.410	0.372	0.360	0.367	0.358	0.319
ensemble catnet	0.900	0.880	0.840	0.814	0.689	0.468	0.356	0.281

TABLE S3 Accuracy rate of top-k regulation relationship of methods on *e.coli* dataset.

Methods	k=50	k=100	k=200	k=500	k=1000	k=2000	k=3000	k=4000
GRINCD	0.206	0.174	0.165	**0.187**	**0.160**	**0.107**	**0.081**	**0.066**
catnet	0.060	0.090	0.080	0.050	0.043	0.038	0.030	0.027
GENLAB	0.280	0.260	**0.235**	0.184	0.127	0.084	0.064	0.052
NARROMI	0.260	0.240	0.165	0.104	0.067	0.049	0.040	0.033
Pearson	0.180	0.160	0.125	0.074	0.044	0.041	0.034	0.030
Stable lasso	0.160	0.130	0.095	0.090	0.058	0.045	0.040	0.035
GRNBOOST2	0.140	0.120	0.145	0.148	0.117	0.077	0.062	0.052
pairwise entropy	0.140	0.090	0.080	0.052	0.047	0.043	0.038	0.031
ppcor	0.140	0.080	0.045	0.022	0.011	0.009	0.008	0.007
BMA	0.000	0.000	0.015	0.026	0.028	0.027	0.026	0.027
PLSNET	0.140	0.120	0.170	0.136	0.105	0.072	0.056	0.046
TIGRESS	**0.320**	**0.270**	0.200	0.126	0.095	0.061	0.050	0.042
CLR	0.160	0.150	0.115	0.136	0.112	0.073	0.056	0.044
PIDC	0.080	0.100	0.125	0.106	0.075	0.054	0.046	0.038
GENIE3	0.060	0.080	0.150	0.168	0.124	0.080	0.059	0.048
Lasso	0.060	0.060	0.060	0.052	0.057	0.054	0.039	0.032
conditional entropy	0.060	0.170	0.155	0.078	0.046	0.033	0.025	0.023
ensemble catnet	0.060	0.100	0.065	0.042	0.037	0.034	0.027	0.025

TABLE S4 Accuracy rate of top-k regulation relationship of methods on *s.cere* dataset.

Methods	k=50	k=100	k=200	k=500	k=1000	k=2000	k=3000	k=4000
GRINCD	0.023	0.022	0.022	0.018	0.015	0.012	0.011	0.010
catnet	0.040	0.030	0.015	0.010	0.008	0.007	0.005	0.005
GENLAB	0.020	0.010	0.005	0.016	0.011	0.010	0.009	0.009
NARROMI	0.040	0.030	0.030	0.012	0.008	0.007	0.005	0.005
Pearson	0.040	0.030	0.020	0.010	0.006	0.004	0.003	0.003
Stable lasso	0.040	0.030	0.015	0.010	0.008	0.008	0.008	0.007
GRNBOOST2	0.020	0.020	0.020	0.018	0.015	0.010	0.008	0.008
pairwise entropy	0.040	0.030	0.020	0.010	0.006	0.005	0.005	0.004
ppcor	0.020	0.010	0.005	0.002	0.001	0.002	0.002	0.003
BMA	0.040	0.040	**0.050**	**0.034**	**0.032**	0.017	0.013	0.011
PLSNET	0.020	0.020	0.020	0.024	0.023	**0.019**	**0.019**	**0.017**
TIGRESS	**0.060**	0.040	0.025	0.014	0.010	0.009	0.009	0.009
CLR	**0.060**	**0.050**	0.045	0.022	0.015	0.009	0.008	0.008
PIDC	0.000	0.000	0.000	0.000	0.001	0.004	0.003	0.003
GENIE3	0.020	0.020	0.020	0.016	0.008	0.009	0.008	0.007
Lasso	0.000	0.000	0.010	0.012	0.006	0.006	0.006	0.006
conditional entropy	0.000	0.000	0.000	0.004	0.006	0.006	0.007	0.007
ensemble catnet	0.040	0.030	0.015	0.010	0.006	0.005	0.004	0.004

TABLE S5 Accuracy rate of top-k regulation relationship of methods on hESC_CTS_500 dataset.

Methods	k=50	k=100	k=200	k=500	k=1000	k=2000	k=3000	k=4000
GRINCD	0.199	**0.211**	**0.215**	**0.216**	**0.217**	**0.217**	**0.213**	**0.207**
catnet	0.100	0.150	0.161	0.178	0.199	0.187	0.167	0.178
NARROMI	0.200	0.130	0.145	0.146	0.146	0.151	0.154	0.160
Pearson	0.200	0.170	0.170	0.166	0.160	0.166	0.161	0.159
GRNBOOST2	0.060	0.030	0.075	0.108	0.131	0.142	0.144	0.147
ppcor	0.080	0.110	0.160	0.156	0.151	0.155	0.159	0.154
PLSNET	0.100	0.080	0.080	0.090	0.094	0.098	0.110	0.113
TIGRESS	**0.220**	0.130	0.095	0.112	0.131	0.137	0.135	0.136
CLR	0.180	0.150	0.190	0.160	0.145	0.145	0.139	0.138
PIDC	0.000	0.000	0.005	0.004	0.004	0.004	0.004	0.004
GENIE3	0.180	0.170	0.140	0.124	0.128	0.119	0.117	0.125
GENLAB	0.209	0.162	0.137	0.161	0.136	0.146	0.138	0.135
Stable lasso	0.197	0.151	0.099	0.110	0.099	0.121	0.123	0.122
pairwise entropy	0.188	0.141	0.107	0.090	0.082	0.099	0.103	0.100
BMA	0.083	0.076	0.086	0.094	0.099	0.105	0.098	0.108
Lasso	0.105	0.087	0.094	0.111	0.112	0.128	0.120	0.120
conditional entropy	0.059	0.086	0.078	0.069	0.069	0.093	0.104	0.112
ensemble catnet	0.175	0.151	0.105	0.104	0.097	0.101	0.096	0.093

TABLE S6 Accuracy rate of top-k regulation relationship of methods on hESC_CTS_1000 dataset.

Methods	k=50	k=100	k=200	k=500	k=1000	k=2000	k=3000	k=4000
GRINCD	0.146	0.160	0.169	**0.190**	**0.200**	**0.203**	**0.203**	**0.202**
catnet	0.110	0.153	0.171	0.171	0.191	0.168	0.157	0.168
NARROMI	0.140	0.130	0.145	0.148	0.136	0.141	0.149	0.150
Pearson	**0.200**	0.150	0.180	0.164	0.160	0.154	0.161	0.162
GRNBOOST2	0.040	0.050	0.065	0.098	0.113	0.144	0.145	0.150
ppcor	0.140	0.110	0.125	0.144	0.149	0.151	0.158	0.157
PLSNET	0.060	0.050	0.060	0.094	0.104	0.112	0.112	0.114
TIGRESS	**0.200**	0.130	0.105	0.120	0.129	0.137	0.139	0.135
CLR	**0.200**	0.160	**0.185**	0.156	0.150	0.146	0.144	0.142
PIDC	0.000	0.000	0.000	0.004	0.002	0.003	0.003	0.003
GENIE3	**0.200**	**0.180**	0.165	0.138	0.126	0.124	0.115	0.118
GENLAB	0.181	0.155	0.118	0.141	0.126	0.136	0.132	0.132
Stable lasso	0.170	0.144	0.085	0.096	0.091	0.113	0.117	0.119
pairwise entropy	0.163	0.135	0.092	0.079	0.076	0.093	0.098	0.097
BMA	0.072	0.073	0.074	0.082	0.091	0.098	0.093	0.105
Lasso	0.091	0.083	0.081	0.098	0.103	0.120	0.114	0.117
conditional entropy	0.051	0.083	0.067	0.061	0.064	0.087	0.099	0.109
ensemble catnet	0.152	0.145	0.090	0.092	0.089	0.095	0.091	0.091

TABLE S7 Accuracy rate of top-k regulation relationship of methods on hESC_NS_500 dataset.

Methods	k=50	k=100	k=200	k=500	k=1000	k=2000	k=3000	k=4000
GRINCD	**0.055**	**0.060**	**0.055**	**0.049**	**0.043**	**0.036**	**0.033**	**0.032**
catnet	0.022	0.041	0.043	0.035	0.035	0.027	0.028	0.025
NARROMI	0.040	**0.060**	0.045	0.032	0.032	0.024	0.023	0.021
Pearson	0.020	0.040	0.035	0.022	0.018	0.020	0.016	0.017
GRNBOOST2	0.020	0.020	0.020	0.024	0.019	0.023	0.022	0.021
ppcor	0.000	0.010	0.015	0.018	0.014	0.017	0.017	0.016
PLSNET	0.000	0.020	0.015	0.018	0.026	0.024	0.023	0.023
TIGRESS	0.040	0.050	0.045	0.044	0.038	0.030	0.028	0.026
CLR	0.000	0.000	0.025	0.044	0.032	0.028	0.022	0.021
PIDC	0.000	0.000	0.005	0.010	0.009	0.012	0.014	0.011
GENIE3	0.040	0.020	0.030	0.024	0.019	0.018	0.021	0.022
GENLAB	0.042	0.041	0.035	0.036	0.027	0.024	0.021	0.021
Stable lasso	0.039	0.038	0.025	0.025	0.020	0.020	0.019	0.019
pairwise entropy	0.038	0.035	0.027	0.020	0.016	0.016	0.016	0.015
BMA	0.017	0.019	0.022	0.021	0.020	0.017	0.015	0.017
Lasso	0.021	0.022	0.024	0.025	0.022	0.021	0.019	0.019
conditional entropy	0.012	0.022	0.020	0.016	0.014	0.015	0.016	0.017
ensemble catnet	0.035	0.038	0.027	0.024	0.019	0.017	0.015	0.014

TABLE S8 Accuracy rate of top-k regulation relationship of methods on hESC_NS_1000 dataset.

Methods	k=50	k=100	k=200	k=500	k=1000	k=2000	k=3000	k=4000
GRINCD	0.045	**0.052**	**0.050**	**0.043**	**0.040**	**0.034**	**0.031**	**0.029**
catnet	0.041	0.041	0.039	0.028	0.035	0.029	0.028	0.021
NARROMI	0.040	0.050	0.040	0.026	0.031	0.022	0.020	0.018
Pearson	0.020	0.040	0.030	0.022	0.017	0.021	0.016	0.015
GRNBOOST2	**0.060**	0.030	0.020	0.020	0.021	0.020	0.019	0.018
ppcor	0.000	0.000	0.000	0.002	0.006	0.009	0.009	0.010
PLSNET	0.020	0.020	0.010	0.028	0.027	0.026	0.022	0.022
TIGRESS	0.040	0.040	**0.050**	0.040	0.033	0.030	0.028	0.025
CLR	0.000	0.000	0.015	0.040	0.028	0.026	0.022	0.019
PIDC	0.000	0.000	0.005	0.006	0.004	0.006	0.008	0.009
GENIE3	0.020	0.050	0.025	0.026	0.021	0.019	0.018	0.021
GENLAB	0.042	0.035	0.032	0.032	0.025	0.023	0.020	0.019
Stable lasso	0.039	0.033	0.023	0.022	0.018	0.019	0.018	0.017
pairwise entropy	0.038	0.031	0.025	0.018	0.015	0.016	0.015	0.014
BMA	0.017	0.016	0.020	0.019	0.018	0.016	0.014	0.015
Lasso	0.021	0.019	0.022	0.022	0.021	0.020	0.017	0.017
conditional entropy	0.012	0.019	0.018	0.014	0.013	0.015	0.015	0.016
ensemble catnet	0.035	0.033	0.024	0.021	0.018	0.016	0.014	0.013

TABLE S9 Accuracy rate of top-k regulation relationship of methods on hESC_STR_500 dataset.

Methods	k=50	k=100	k=200	k=500	k=1000	k=2000	k=3000	k=4000
GRINCD	**0.103**	0.090	0.077	0.072	0.068	**0.064**	**0.062**	**0.060**
catnet	0.081	0.061	0.099	0.081	0.067	0.049	0.058	0.043
NARROMI	0.080	**0.110**	0.075	0.076	0.069	0.055	0.049	0.044
Pearson	0.060	0.050	0.060	0.048	0.036	0.032	0.028	0.025
GRNBOOST2	0.040	0.050	0.065	0.058	0.050	0.051	0.050	0.047
ppcor	0.040	0.040	0.045	0.026	0.023	0.019	0.019	0.019
PLSNET	0.040	0.050	0.050	0.058	0.055	0.047	0.041	0.040
TIGRESS	0.060	0.090	0.095	0.100	0.077	0.060	0.055	0.052
CLR	0.080	0.060	**0.100**	**0.102**	**0.079**	0.063	0.052	0.047
PIDC	0.040	0.040	0.035	0.034	0.026	0.032	0.032	0.028
GENIE3	0.020	0.020	0.040	0.044	0.047	0.045	0.041	0.043
GENLAB	0.072	0.074	0.064	0.076	0.050	0.043	0.040	0.039
Stable lasso	0.068	0.069	0.046	0.052	0.036	0.036	0.036	0.035
pairwise entropy	0.065	0.065	0.050	0.042	0.030	0.029	0.030	0.029
BMA	0.028	0.035	0.040	0.044	0.036	0.031	0.029	0.031
Lasso	0.036	0.040	0.044	0.052	0.041	0.038	0.035	0.035
conditional entropy	0.020	0.040	0.036	0.033	0.025	0.027	0.030	0.032
ensemble catnet	0.060	0.069	0.049	0.049	0.035	0.030	0.028	0.027

TABLE S10 Accuracy rate of top-k regulation relationship of methods on hESC_STR_1000 dataset.

Methods	k=50	k=100	k=200	k=500	k=1000	k=2000	k=3000	k=4000
GRINCD	**0.096**	0.086	0.073	0.065	0.064	0.060	**0.056**	**0.054**
catnet	0.070	0.055	0.067	0.059	0.057	0.051	0.049	0.031
NARROMI	0.060	**0.100**	**0.080**	0.062	0.055	0.046	0.041	0.042
Pearson	0.040	0.050	0.045	0.048	0.036	0.028	0.028	0.025
GRNBOOST2	0.060	0.040	0.050	0.046	0.045	0.054	0.047	0.046
ppcor	0.000	0.020	0.015	0.008	0.010	0.009	0.010	0.010
PLSNET	0.020	0.040	0.040	0.048	0.051	0.047	0.041	0.039
TIGRESS	0.060	0.070	**0.080**	**0.090**	**0.079**	**0.068**	**0.056**	0.053
CLR	0.040	0.020	0.040	0.070	0.065	0.056	0.048	0.045
PIDC	0.000	0.000	0.020	0.020	0.015	0.015	0.016	0.020
GENIE3	0.000	0.020	0.030	0.036	0.038	0.035	0.038	0.040
GENLAB	0.067	0.068	0.051	0.067	0.050	0.046	0.036	0.035
Stable lasso	0.063	0.063	0.037	0.046	0.036	0.038	0.032	0.032
pairwise entropy	0.060	0.059	0.040	0.037	0.030	0.031	0.027	0.026
BMA	0.026	0.032	0.032	0.039	0.036	0.033	0.026	0.028
Lasso	0.034	0.036	0.035	0.046	0.041	0.040	0.032	0.031
conditional entropy	0.019	0.036	0.029	0.029	0.025	0.029	0.027	0.029
ensemble catnet	0.056	0.063	0.039	0.044	0.035	0.032	0.025	0.024

TABLE S11 Accuracy rate of top-k regulation relationship of methods on mDC_CTS_500 dataset.

Methods	k=50	k=100	k=200	k=500	k=1000	k=2000	k=3000	k=4000
GRINCD	0.097	0.099	0.098	**0.084**	0.070	0.060	0.056	0.054
catnet	0.035	0.046	0.055	0.066	**0.670**	0.051	0.049	0.043
NARROMI	0.100	0.100	**0.100**	0.064	0.069	0.062	0.058	0.056
Pearson	0.000	0.060	0.040	0.048	0.063	0.060	0.056	0.053
GRNBOOST2	0.060	0.050	0.040	0.060	0.056	0.055	0.049	0.050
ppcor	0.040	0.070	0.065	0.062	0.053	0.048	0.047	0.046
PLSNET	0.120	0.100	0.075	0.078	0.072	0.059	0.052	0.054
TIGRESS	**0.200**	**0.140**	0.095	0.070	0.066	**0.063**	**0.062**	**0.061**
CLR	0.060	0.050	0.050	0.056	0.061	0.058	0.050	0.050
PIDC	0.000	0.000	0.010	0.004	0.002	0.002	0.002	0.002
GENIE3	0.080	0.120	0.095	0.078	0.067	**0.063**	0.059	0.055
GENLAB	0.139	0.095	0.064	0.063	0.421	0.042	0.040	0.040
Stable lasso	0.131	0.088	0.046	0.043	0.306	0.035	0.036	0.036
pairwise entropy	0.126	0.082	0.050	0.035	0.254	0.029	0.030	0.029
BMA	0.055	0.044	0.040	0.036	0.305	0.030	0.029	0.032
Lasso	0.070	0.051	0.044	0.043	0.345	0.037	0.035	0.035
conditional entropy	0.039	0.050	0.036	0.027	0.214	0.027	0.030	0.033
ensemble catnet	0.117	0.088	0.049	0.041	0.300	0.029	0.028	0.028

TABLE S12 Accuracy rate of top-k regulation relationship of methods on mDC_CTS_1000 dataset.

Methods	k=50	k=100	k=200	k=500	k=1000	k=2000	k=3000	k=4000
GRINCD	0.113	0.102	0.091	0.070	0.058	0.048	0.045	0.043
catnet	0.091	0.081	0.041	0.051	0.044	0.033	0.037	0.042
NARROMI	0.020	0.030	0.060	0.060	0.062	**0.068**	**0.061**	0.056
Pearson	0.000	0.050	0.045	0.044	0.058	0.057	0.059	0.055
GRNBOOST2	0.080	0.080	0.055	0.062	0.052	0.054	0.050	0.052
ppcor	0.100	0.090	0.070	0.052	0.052	0.048	0.044	0.044
PLSNET	0.100	**0.130**	**0.110**	0.078	0.069	0.057	0.056	0.053
TIGRESS	**0.200**	**0.130**	**0.110**	**0.088**	0.066	0.061	0.059	**0.060**
CLR	0.080	0.060	0.055	0.058	0.060	0.059	0.052	0.051
PIDC	0.000	0.000	0.000	0.004	0.002	0.002	0.001	0.001
GENIE3	0.080	0.080	0.085	0.082	**0.077**	0.064	0.059	0.055
GENLAB	0.139	0.088	0.070	0.065	0.048	0.046	0.040	0.039
Stable lasso	0.131	0.082	0.051	0.045	0.035	0.038	0.035	0.035
pairwise entropy	0.126	0.077	0.055	0.037	0.029	0.031	0.030	0.029
BMA	0.055	0.041	0.044	0.038	0.035	0.033	0.028	0.031
Lasso	0.070	0.047	0.048	0.045	0.040	0.040	0.034	0.035
conditional entropy	0.039	0.047	0.040	0.028	0.025	0.029	0.030	0.032
ensemble catnet	0.117	0.082	0.054	0.043	0.034	0.032	0.027	0.027

TABLE S13 Accuracy rate of top-k regulation relationship of methods on mDC_NS_500 dataset.

Methods	k=50	k=100	k=200	k=500	k=1000	k=2000	k=3000	k=4000
GRINCD	0.244	0.198	0.157	**0.110**	**0.081**	**0.059**	**0.049**	**0.043**
catnet	0.170	0.120	0.130	0.098	0.078	0.054	0.045	0.041
NARROMI	0.080	0.040	0.035	0.032	0.024	0.020	0.019	0.019
Pearson	0.000	0.000	0.005	0.008	0.014	0.014	0.015	0.015
GRNBOOST2	0.060	0.060	0.045	0.034	0.032	0.031	0.026	0.024
ppcor	0.040	0.020	0.020	0.022	0.020	0.026	0.024	0.024
PLSNET	0.220	0.180	0.120	0.090	0.061	0.046	0.039	0.036
TIGRESS	0.180	0.150	0.105	0.068	0.059	0.045	0.036	0.034
CLR	**0.360**	**0.270**	**0.165**	0.096	0.065	0.048	0.039	0.032
PIDC	0.080	0.120	0.125	0.078	0.047	0.031	0.025	0.022
GENIE3	0.180	0.190	0.150	0.082	0.060	0.042	0.038	0.034
GENLAB	0.251	0.182	0.105	0.082	0.051	0.040	0.032	0.028
Stable lasso	0.236	0.170	0.076	0.056	0.037	0.033	0.028	0.025
pairwise entropy	0.226	0.159	0.082	0.046	0.031	0.027	0.024	0.021
BMA	0.099	0.085	0.066	0.048	0.037	0.029	0.023	0.022
Lasso	0.126	0.098	0.072	0.057	0.042	0.035	0.028	0.025
conditional entropy	0.071	0.097	0.060	0.035	0.026	0.025	0.024	0.023
ensemble catnet	0.211	0.170	0.081	0.053	0.036	0.027	0.022	0.019

TABLE S14 Accuracy rate of top-k regulation relationship of methods on mDC_NS_1000 dataset.

Methods	k=50	k=100	k=200	k=500	k=1000	k=2000	k=3000	k=4000
GRINCD	0.239	0.189	0.147	0.101	**0.074**	**0.054**	**0.046**	**0.040**
catnet	0.091	0.153	0.134	0.097	0.066	0.044	0.042	0.038
NARROMI	0.060	0.040	0.045	0.032	0.026	0.024	0.023	0.021
Pearson	0.000	0.000	0.005	0.004	0.014	0.013	0.014	0.016
GRNBOOST2	0.040	0.040	0.050	0.038	0.035	0.032	0.027	0.025
ppcor	0.000	0.000	0.020	0.036	0.027	0.023	0.020	0.019
PLSNET	0.140	0.140	0.115	0.080	0.057	0.046	0.038	0.034
TIGRESS	0.180	0.170	0.120	0.084	0.061	0.045	0.036	0.033
CLR	**0.360**	**0.280**	**0.185**	**0.112**	0.073	0.050	0.040	0.035
PIDC	0.020	0.070	0.095	0.076	0.048	0.029	0.022	0.019
GENIE3	0.160	0.170	0.125	0.082	0.061	0.048	0.041	0.035
GENLAB	0.251	0.189	0.118	0.083	0.047	0.036	0.030	0.026
Stable lasso	0.236	0.176	0.085	0.057	0.034	0.030	0.027	0.024
pairwise entropy	0.226	0.165	0.092	0.047	0.028	0.025	0.022	0.019
BMA	0.099	0.088	0.074	0.049	0.034	0.026	0.021	0.021
Lasso	0.126	0.101	0.081	0.058	0.038	0.032	0.026	0.023
conditional entropy	0.071	0.101	0.067	0.036	0.024	0.023	0.022	0.022
ensemble catnet	0.211	0.176	0.090	0.054	0.033	0.025	0.021	0.018

TABLE S15 Accuracy rate of top-k regulation relationship of methods on mDC_STR_500 dataset.

Methods	k=50	k=100	k=200	k=500	k=1000	k=2000	k=3000	k=4000
GRINCD	0.322	0.267	0.213	0.143	0.102	**0.073**	**0.060**	**0.053**
catnet	0.224	0.216	0.178	0.136	0.091	0.062	0.043	0.039
NARROMI	0.180	0.150	0.110	0.076	0.057	0.043	0.040	0.038
Pearson	0.000	0.000	0.015	0.024	0.034	0.031	0.032	0.030
GRNBOOST2	0.100	0.100	0.075	0.058	0.054	0.043	0.042	0.042
ppcor	0.120	0.080	0.050	0.046	0.042	0.035	0.031	0.033
PLSNET	0.420	0.310	0.220	0.130	0.091	0.062	0.053	0.046
TIGRESS	0.340	0.240	0.170	0.112	0.091	0.065	0.058	0.052
CLR	**0.640**	**0.460**	**0.315**	**0.186**	**0.117**	0.070	0.051	0.044
PIDC	0.320	0.320	0.255	0.142	0.085	0.054	0.041	0.035
GENIE3	0.440	0.340	0.210	0.134	0.091	0.065	0.052	0.046
GENLAB	0.446	0.311	0.201	0.138	0.074	0.049	0.039	0.035
Stable lasso	0.420	0.289	0.145	0.094	0.053	0.041	0.035	0.031
pairwise entropy	0.402	0.271	0.157	0.077	0.044	0.033	0.029	0.026
BMA	0.176	0.145	0.126	0.081	0.053	0.035	0.028	0.028
Lasso	0.223	0.167	0.137	0.096	0.060	0.043	0.034	0.031
conditional entropy	0.125	0.165	0.115	0.060	0.037	0.031	0.029	0.029
ensemble catnet	0.374	0.289	0.154	0.090	0.052	0.034	0.027	0.024

TABLE S16 Accuracy rate of top-k regulation relationship of methods on mDC_STR_1000 dataset.

Methods	k=50	k=100	k=200	k=500	k=1000	k=2000	k=3000	k=4000
GRINCD	0.299	0.239	0.185	0.123	0.089	0.063	0.052	**0.046**
catnet	0.218	0.245	0.181	0.115	0.076	0.065	0.043	0.044
NARROMI	0.160	0.150	0.105	0.066	0.048	0.042	0.039	0.037
Pearson	0.000	0.000	0.015	0.020	0.029	0.031	0.029	0.029
GRNBOOST2	0.080	0.100	0.070	0.052	0.054	0.044	0.038	0.037
ppcor	0.040	0.080	0.065	0.074	0.053	0.039	0.037	0.033
PLSNET	0.360	0.320	0.215	0.128	0.088	0.059	0.048	0.043
TIGRESS	0.360	0.240	0.185	0.102	0.084	0.063	**0.054**	**0.046**
CLR	**0.660**	**0.440**	**0.325**	**0.178**	**0.118**	**0.072**	0.053	**0.046**
PIDC	0.200	0.230	0.200	0.134	0.079	0.049	0.037	0.031
GENIE3	0.380	0.300	0.210	0.132	0.091	0.063	0.053	**0.046**
GENLAB	0.459	0.297	0.207	0.132	0.074	0.048	0.035	0.030
Stable lasso	0.433	0.276	0.150	0.090	0.054	0.040	0.031	0.027
pairwise entropy	0.415	0.259	0.162	0.074	0.045	0.033	0.026	0.022
BMA	0.182	0.139	0.130	0.077	0.054	0.035	0.025	0.024
Lasso	0.230	0.159	0.142	0.092	0.061	0.042	0.030	0.027
conditional entropy	0.129	0.158	0.118	0.057	0.038	0.031	0.026	0.025
ensemble catnet	0.386	0.277	0.159	0.086	0.053	0.034	0.024	0.021

TABLE S17 Results of different node embeddings and causal modules on *in silico* dataset.

Combinations	AUROC	AUPR	confidence score	EPR
GRINCD	0.725	0.169	8.465	18.306
GraphSAGE+IGCI	0.693	0.166	5.410	15.896
GraphSAGE+RECI	0.704	0.156	7.958	13.853
GCN+ANM	0.724	0.165	7.759	15.369
GCN+IGCI	0.748	0.149	6.890	16.513
GCN+RECI	0.661	0.157	9.078	13.884
GAT+ANM	0.674	0.165	9.003	17.766
GAT+IGCI	0.684	0.135	5.427	15.009
GAT+RECI	0.702	0.163	7.825	16.160

TABLE S18 Results of different node embeddings and causal modules on *e.coli* dataset.

Combinations	AUROC	AUPR	confidence score	EPR
GRINCD	0.626	0.111	24.026	76.447
GraphSAGE+IGCI	0.618	0.091	22.136	74.780
GraphSAGE+RECI	0.591	0.112	20.623	66.220
GCN+ANM	0.622	0.076	19.623	73.173
GCN+IGCI	0.604	0.100	22.863	69.332
GCN+RECI	0.565	0.105	22.739	62.859
GAT+ANM	0.595	0.109	23.351	72.691
GAT+IGCI	0.621	0.094	22.739	67.472
GAT+RECI	0.612	0.109	18.026	69.965

TABLE S19 Results of different node embeddings and causal modules on *s.cere* dataset.

Combinations	AUROC	AUPR	confidence score	EPR
GRINCD	0.571	0.024	60.374	5.076
GraphSAGE+IGCI	0.560	0.012	53.149	4.858
GraphSAGE+RECI	0.538	0.027	54.711	5.336
GCN+ANM	0.554	0.018	58.688	2.917
GCN+IGCI	0.547	0.028	63.841	4.826
GCN+RECI	0.573	0.013	51.805	5.891
GAT+ANM	0.554	0.029	60.108	4.895
GAT+IGCI	0.562	0.010	49.474	3.121
GAT+RECI	0.566	0.017	51.684	4.620

TABLE S20 Results of different node embeddings and causal modules on *s.aureus* dataset.

Combinations	AUROC	AUPR	confidence score	EPR
GRINCD	0.654	0.071	25.684	25.884
GraphSAGE+IGCI	0.585	0.061	20.132	24.186
GraphSAGE+RECI	0.624	0.065	22.479	22.277
GCN+ANM	0.605	0.069	25.797	20.458
GCN+IGCI	0.628	0.069	18.963	21.669

GCN+RECI	0.631	0.052	23.073	22.969
GAT+ANM	0.666	0.070	28.072	20.170
GAT+IGCI	0.609	0.060	15.360	21.550
GAT+RECI	0.617	0.070	19.431	19.085

TABLE S22 Comparison of different ANM implementations on *in silico* dataset.

Methods	AUPR	AUROC	confidence score	EPR
GRINCD	0.169	0.725	8.465	18.306
ANM by NN	0.170	0.694	8.005	19.452

TABLE S22 Comparison of different ANM implementations on *e.coli* dataset.

Methods	AUPR	AUROC	confidence score	EPR
GRINCD	0.111	0.626	24.026	76.447
ANM by NN	0.103	0.589	25.124	60.561

TABLE S23 Comparison of different ANM implementations on *s.cere* dataset.

Methods	AUPR	AUROC	confidence score	EPR
GRINCD	0.024	0.571	60.374	5.076
ANM by NN	0.025	0.569	61.672	3.671

TABLE S24 Comparison of different ANM implementations on *s.aureus* dataset.

Methods	AUPR	AUROC	confidence score	EPR
GRINCD	0.071	0.654	25.684	25.884
ANM by NN	0.069	0.641	24.178	30.773
